# Gestational age and trajectories of body mass index and height from birth through adolescence in the Danish National Birth Cohort

**DOI:** 10.1038/s41598-023-30123-y

**Published:** 2023-02-26

**Authors:** Johan L. Vinther, Claus T. Ekstrøm, Thorkild I. A. Sørensen, Luise Cederkvist, Deborah A. Lawlor, Anne-Marie Nybo Andersen

**Affiliations:** 1 Section of Epidemiology, Department of Public Health, University of Copenhagen, Bartholinsgade 6Q, 2nd Fl., 1356 Copenhagen, Denmark; 2 Section of Biostatistics, Department of Public Health, University of Copenhagen, Copenhagen, Denmark; 3 Novo Nordisk Foundation Center for Basic Metabolic Research, Faculty of Health and Medical Sciences, University of Copenhagen, Copenhagen, Denmark; 4 Population Health Science, Bristol Medical School, Bristol, BS8 2BN UK; 5 MRC Integrative Epidemiology Unit at the University of Bristol, Bristol, BS8 2BN UK

**Keywords:** Cardiology, Health care, Medical research, Risk factors

## Abstract

Preterm birth is associated with smaller body dimensions at birth. The impact on body size in later life, measured by body mass index (BMI) and height, remains unclear. A prospective register-based cohort study with 62,625 singletons from the Danish National Birth Cohort born 1996–2003 for whom information on gestational age (GA) at birth, length or weight at birth, and at least two growth measurements scheduled at the ages of 5 and 12 months, and 7, 11 and 18 years were available. Linear mixed effects with splines, stratified by sex, and adjusted for confounders were used to estimate standardised BMI and height. GA was positively associated with BMI in infancy, but differences between preterm and term children declined with age. By age 7, preterm children had slightly lower BMI than term children, whereas no difference was observed by adolescence (mean difference in BMI z-score − 0.28 to 0.15). GA was strongly associated with height in infancy, but mean differences between individuals born preterm and term declined during childhood. By adolescence, the most preterm individuals remained shorter than their term peers (mean difference in height z-score from − 1.00 to − 0.28). The lower BMI in preterm infants relative to term infants equalizes during childhood, such that by adolescence there is no clear difference. Height is strongly positively associated with GA in early childhood, whilst by end of adolescence individuals born preterm remain slightly shorter than term peers.

## Introduction

Preterm birth is one of the leading causes of perinatal mortality and morbidity^1^, and some evidence suggest associations with long-term health and social outcomes^2,3^.

Exposures during fetal life may influence postnatal growth and cardio-metabolic health^4,5^. Much literature has demonstrated that birth weight associates with postnatal body mass index (BMI) with positive linear J and U-shaped associations reported^6–8^. Birth weight is strongly related to gestational age at birth (GA)^9^, but the extent to which the association between birth weight and later BMI reflects differences in GA is unclear^10^.

Some studies have investigated the associations between GA and BMI and, respectively, height^3,11–20^ and reported positive associations in childhood^13,21^, while findings are mixed in adulthood^20,22^. However, wide variations in study design, mode of data collection, and use of covariates in the studies making it difficult to evaluate the evidence^23^. Some studies assessed gestational duration as a dichotomous variable reporting all preterm children in same exposure group^3,24^, while others compared growth of extremely preterm with term, which may hide important differences by degree of preterm^3^. We identified only two previous publications that reported associations with BMI and height for subgroups of preterm and term born children^13,25^.

In addition, majority of studies have examined the association without repeat measurements of BMI or height; hence, evidence on the longitudinal association between gestational age and BMI and height is limited. A life-course approach that enables tracking of BMI and height during infancy, childhood and in adolescence by where individuals have reached their maximum height and a stabilized BMI may add to the evidence^26^.

The aim of this study was to examine longitudinal associations between length of gestation at birth and trajectories of BMI and height, respectively, from birth through adolescence. We explored sex-specific associations due to natural differences in growth^27^.

## Methods

### Study design

This is a longitudinal study using data from the Danish National Birth Cohort (DNBC), which includes information on 96,822 live-born children and their mothers^28^. Baseline information was planned to be collected at 12 and 30 weeks gestation, and follow-ups with information on height and weight were planned to be collected at 18 months and 7, 11 and 18 years. Information is linked with nation-wide registry data at Statistics Denmark^29–32^ as described below. Details of the DNBC are given elsewhere^28^.

### Study population

**Figure 1:**
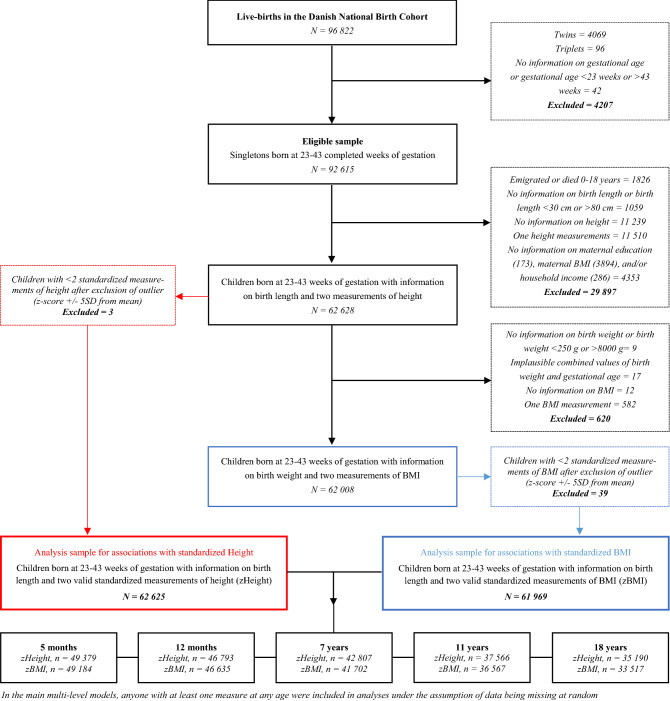
Flow chart of eligibility and inclusion in the study population in the study.

Eligible for this study was any live-born singleton in the DNBC with a gestational age of 23–43 completed weeks at birth (*n* = 92,615) (Fig. 1). We excluded individuals who emigrated or died (*n* = 1826) during follow-up, and excluded individuals with less than two growth measurements after birth and without information on potential confounders: maternal education (*n* = 173), maternal pre-pregnancy BMI (*n* = 3894) and/or household income (*n* = 286). Before defining two specific populations for analysis, individuals with missing information on birth length (*n* = 374) or birth length < 30 cm or > 80 cm (*n* = 685), and with less than two measurements of height after birth (*n* = 22,749) were excluded.

In the sample for height, we excluded three additional individuals with height values ± 5 standard deviations from the mean following recommendations from Vidmar et al. leaving 62,625 individuals for analysis^33^.

In the sample for BMI, we excluded an additional 620 individuals without information on birth weight or with implausible combined values of birth weight and GA (*n* = 26), and individuals with less than two measurements of BMI (*n* = 594). Lastly, 39 individuals with BMI values ± 5 standard deviations from the mean was excluded, which left 61,969 individuals for analysis^33^.

### Gestational age

Information on GA in days was obtained from the Danish Medical Birth Register^29^. The GA was reported to the register by the midwife at birth, based on results of ultra sound scans (at the time not part of recommended care, however made on almost 80% of women around week 18), anamnestic information about LMP and cycle length and regularity, and the clinical judgement of the child. GA was converted to completed weeks and categorized into seven groups: extremely preterm (23–27 weeks), very preterm (28–31 weeks), moderately preterm (32–33 weeks), late preterm (34–36 weeks), early term (37–38 weeks), term (39–41 weeks), and post term (42–43 weeks).

### Body mass index and height

Information on birth weight and birth length was derived from the Danish Medical Birth Register^29^. Mothers were interviewed when the child was around 18 months and asked to report the weight and height registered in the ‘Child’s book’ at the preventive child examinations in general practice at scheduled ages of 5 and 12 months (measured at 1–22 months). These were used in the analyses together with height and weight reported by mothers at questionnaire-based follow-ups, scheduled at ages of 7 (measured at 5–7 years) and 11 years (measured at 10–14 years)^28^. At the 18 year follow-up (in practice at 17–19 years), height and weight were self-reported by the adolescent.

For each child, standardized scores (z-scores) of BMI and length at birth, and BMI and height after birth to age 19 years (228 months) were calculated separately for boys and girls. Standardization was done internally using 1-month categories.

### Confounders

Confounders were selected a priori based on previous evidence^2,34–36^. Information on maternal height (cm), maternal pre-pregnancy BMI (kg/m^2^), and maternal smoking during pregnancy (yes/no) was taken from questionnaires at 12 and 30 weeks of gestation. Information on maternal age at delivery (continuous in years); gestational diabetes, ICD-10: O24 (yes/no); gestational hypertension, ICD-10: O13 (yes/no); and pre-eclampsia, ICD-10: O14 (yes/no) was derived from Danish Medical Birth Registry. Maternal education was obtained from the Danish Population’s Education Register and operationalised as highest ongoing or completed education at child’s birth according to international classification standards^37^: low [ISCED-2011: 0–2], medium [ISCED-2011: 3–4], high [ISCED-2011: 5–8]). Household income was based on disposable household income extracted from the Income Statistics Register. The variable was divided by an equivalence factor according to household size (available at http://www.oecd.org) and recoded into internal quantiles per year. Birth weight was perceived as an intermediate variable on the causal pathway, hence not included in the models^38^.

### Statistical analysis

Linear-mixed effects models were used to estimate the association between GA in categories and BMI and height z-scores, respectively, from birth through age 19 years (228 months) while adjusting for confounders^39^. Analyses were performed by sex due to natural differences in growth trajectories^27^.

Linear splines are a commonly used type of regression spline for repeated measurements of non-linear growth trajectories^40^. Therefore, to best approximate the relationship between GA and the standardized growth measurements, we included linear splines in our models with four internal knots (5, 12, 85 and 134 months) and two boundary knots (at birth and 228 months). The knots were chosen a priori based on amount of available data^40^.

The linear mixed-effects model with linear splines (LME) accommodated for repeated measurements from the same child, without imposing any structure on the correlations among the time points using an unstructured covariance matrix. Also, in the LME models we accounted for clustering of children with same mothers. Age was set to 0 months at birth and included as a continuous variable, and the actual age in months at measurements of BMI and height was included in the models.

Furthermore, nearly one-third of individuals was excluded from the eligible sample (32%) (S1 Table), thus by adding inverse probability weights (IPWs) to the LME models we sought to remedy bias from this selection. The IPWs were based on variables that predicted selection into our analysis sample: sex, maternal age, maternal education, maternal smoking during pregnancy, maternal pre-pregnancy BMI, household income, parity, and caesarean section^41^.

The fitted LME models were used to predict sex-specific mean BMI and height z-scores by categories of GA with 95% confidence intervals (CIs) at six ages corresponding to the data collections: birth, 5 months, 1 year, 7 years, 11 years, and 18 years. For prediction, we used the following: mean maternal age at delivery (31.2 years), mean maternal pre-pregnancy BMI (23.5 kg/m^2^), mean maternal height (1.69 m), maternal education (high), household income (4th quartile), maternal smoking during pregnancy (no), gestational diabetes (no), gestational hypertension (no), and preeclampsia (no).

We performed sensitivity analyses (S3–S6 Tables), including a model only adjusting for sociodemographic variables, and models without adjustment of covariates, IPW and clustering.

Statistical analyses were carried out using the statistical software R version 4.0^42^ and packages for ‘nlme’ and ‘splines’^43^.

### Ethics approval

All methods were carried out in accordance with relevant guidelines and regulations. The DNBC data collection has been approved by the Regional Scientific Ethical Committee for the Municipalities of Copenhagen and Frederiksberg, and the Danish Data Protection Agency. Informed consent for study participation was obtained from the mother upon enrolment, and confirmed by the adolescent at age 18 years. Approval of the study was obtained from the Danish Data Protection Agency through the joint notification of The Faculty of Health and Medical Sciences at The University of Copenhagen (SUND-2017-09) and the study has furthermore been approved by the DNBC Steering Committee.

## Results

### Descriptive statistics

Baseline characteristics differed across categories of GA in the analysis sample (Table 1). All categories of preterm infants, relative to term, were more likely to have mothers with low educational level, who smoked during pregnancy, were diagnosed with preeclampsia or gestational hypertension, and delivered by caesarean section. Mean age at timing of measurements for height and weight was similar across categories of GA at all data collections, whereas mean BMI (kg/m^2^) was similar for preterm and term individuals after age 5 months, whilst height was consistently lower for preterm individuals throughout childhood and adolescence (S2 Table).

**Table 1:** Selected characteristics of the study population from children born in the Danish National Birth Cohort (1996–2003), n = 62,625.

	Extremely preterm (23–27 weeks)	Very preterm (28–31 weeks)	Moderately preterm (32–33 weeks)	Late preterm (34–36 weeks)	Early term (37–38 weeks)	Term (39–41 weeks)	Post term (42–43 weeks)	Total (23–43 weeks)
Total N of children (%)	34 (0.1%)	160 (0.3%)	292 (0.5%)	2019 (3.2%)	9463 (15.1%)	45,139 (72.1%)	5518 (8.8%)	62,625 (100%)
Sex (%), female	56.2%	48.7%	43.4%	47.1%	47.6%	50.3%	47.9%	49.8%
Gestational age, mean (SD), days	187 (6)	213 (8)	232 (4)	251 (6)	267 (4)	283 (6)	296 (2)	280 (11.5)
Gestational age, mean (SD), weeks	26.2 (0.8)	30.0 (1.0)	32.7 (0.5)	35.4 (0.8)	37.7 (0.4)	40.0 (0.8)	42.0 (0.2)	39.6 (1.7)
Birth weight, mean (SD), g	937 (215)	1528 (680)	2053 (438)	2755 (515)	3323 (477)	3682 (470)	3894 (485)	3602 (546)
Maternal age at delivery, mean (SD), years	32.4 (5)	30.6 (4)	30.8 (5)	30.7 (4)	31.3 (4)	31.1 (4)	31.0 (4)	31.1 (4)
Maternal smoking in pregnancy (%)	29.4%	31.9%	32.9%	26.7%	25.3%	24.1%	24.7%	24.4%
Maternal height, mean (SD), m	1.68 (0.05)	1.68 (0.06)	1.68 (0.06)	1.68 (0.06)	1.68 (0.06)	1.69 (0.06)	1.69 (0.06)	1.69 (0.1)
Maternal pre-pregnancy BMI, mean	23.0	24.1	23.7	23.7	23.6	23.5	24.1	23.6
Maternal pre-pregnancy BMI, (%)								
< 18.5 kg/m^2^	11.8%	3.8%	3.8%	4.9%	5.1%	4.1%	3.1%	4.2%
18.5–25.0 kg/m^2^	67.6%	63.7%	68.5%	65.8%	66.9%	69.3%	64.9%	68.4%
> 25.0 kg/m^2^	20.6%	32.5%	27.7%	29.4%	27.9%	26.6%	32.0%	27.4%
Maternal highest or ongoing education (%)^a^								
Low	20.6%	10.6%	12.7%	10.3%	10.4%	8.9%	8.4%	9.1%
Medium	32.4%	46.9%	43.2%	46.4%	43.5%	42.1%	42.4%	42.5%
High	47.1%	42.5%	44.2%	43.3%	46.1%	49.0%	49.2%	48.4%
Equivalised income, quartiles (%)								
1 (lowest)	20.6%	21.2%	24.0%	23.0%	21.7%	21.9%	22.4%	22.0%
2	26.5%	23.8%	26.0%	23.5%	25.2%	25.8%	24.9%	25.5%
3	35.3%	28.1%	24.0%	25.7%	26.4%	26.1%	27.1%	26.2%
4 (highest)	17.6%	26.9%	26.0%	27.8%	26.7%	26.2%	25.6%	26.3%
Gestational diabetes (%)^b^	0.0%	0.0%	–	1.8%	1.9%	0.6%	0.1%	0.8%
Gestational hypertension (%)	0.0%	8.8%	10.6%	8.5%	6.4%	4.5%	4.1%	4.9%
Preeclampsia (%)	11.8%	20.6%	17.1%	8.4%	3.9%	1.7%	0.8%	2.3%
Delivery by C-section (%)	54.5%	73.2%	49.3%	31.4%	27.5%	10.7%	16.9%	14.8%

*SD* standard deviation, *BMI* body mass index, *C-section* caesarean section.

^a^ISCED 11 is used for classification of maternal educational level.

^b^For moderately preterm, information on gestational diabetes was omitted due to disclosure control and low numbers (< 5) in cell.

### Trajectories of predicted BMI z-score by categories of GA

For extremely, very, moderately and late preterm children, the mean BMI z-score increased noticeably in the first year of life, with catch-up growth (gain in z-score of > 0.67) in preterm infants between birth and 5 months (Table 2, S1 Fig.)^44^. Accordingly, mean differences in BMI z-score between preterm and term infants attenuated in the first 12 months, though BMI remained lower in preterm than term.

**Table 2:** Predicted mean BMI z-score from birth to age 18 years by category of gestational age, *n* = 61,969 (estimated from LME-model).

Age	Standardized mean BMI z-score by category of gestational age, and mean differences in BMI z-score compared to full term reported with 95% confidence interval (CI)							
	N	Extremely preterm 23–27 weeks	Very preterm 28–31 weeks	Moderately preterm 32–33 weeks	Late preterm 34–36 weeks	Early term 37–38 weeks	Term 39–41 weeks	Post term 42–43 weeks
Boys								
Birth	31,127							
Mean (95% CI)		− 4.90 (− 5.67, − 4.12)	− 3.92 (− 4.16, − 3.67)	− 2.84 (− 2.99, − 2.69)	− 1.62 (− 1.68, − 1.56)	− 0.57 (− 0.59, − 0.54)	0.11 (0.09, 0.12)	0.48 (0.44, 0.51)
Mean difference (95% CI)		− 5.00 (− 6.16, − 3.85)	4.02 (− 4.39, − 3.65)	− 2.95 (− 3.18, − 2.72)	− 1.73 (− 1.81, − 1.64)	− 0.67 (− 0.72, − 0.63)	Ref.	0.37 (0.32, 0.43)
5 months	24,948							
Mean (95% CI)		− 0.47 (− 1.80, 0.86)	− 0.80 (− 1.09, − 0.52)	− 0.22 (− 0.40, − 0.05)	− 0.08 (− 0.15, − 0.02)	− 0.04 (− 0.08, − 0.01)	− 0.06 (− 0.08, − 0.04)	− 0.06 (− 0.10, − 0.02)
Mean difference (95% CI)		− 0.40 (− 2.40, 1.60)	− 0.74 (− 1.17, − 0.31)	− 0.16 (− 0.43, 0.10)	− 0.02 (− 0.12, 0.08)	0.02 (− 0.03, 0.07)	Ref.	0.01 (− 0.06, 0.07)
12 months	23,634							
Mean (95% CI)		− 0.84 (− 1.73, 0.04)	− 0.46 (− 0.74, − 0.19)	− 0.04 (− 0.21, 0.14)	− 0.15 (− 0.22, − 0.09)	− 0.06 (− 0.09, − 0.03)	− 0.06 (− 0.08, − 0.04)	− 0.03 (− 0.07, 0.01)
Mean difference (95% CI)		− 0.78 (− 2.12, 0.55)	− 0.40 (− 0.82, 0.01)	0.02 (− 0.24, 0.29)	− 0.09 (− 0.19, 0.00)	− 0.00 (− 0.05, 0.05)	Ref.	0.03 (− 0.03, 0.09)
85 months (7 years)	21,257							
Mean (95% CI)		− 0.23 (− 1.10, 0.63)	− 0.44 (− 0.73, − 0.15)	− 0.24 (− 0.43, − 0.06)	− 0.11 (− 0.18, − 0.04)	− 0.08 (− 0.11, − 0.04)	− 0.07 (− 0.09, − 0.05)	− 0.01 (− 0.05, 0.03)
Mean difference (95% CI)		− 0.17 (− 1.47, 1.14)	− 0.37 (− 0.80, 0.06)	− 0.18 (− 0.45, 0.10)	− 0.04 (− 0.14, 0.07)	− 0.01 (− 0.06, 0.04)	Ref.	0.06 (− 0.01, 0.12)
134 months (11 years)	18,049							
Mean (95% CI)		− 0.16 (− 1.25, 0.93)	0.08 (− 0.26, 0.42)	− 0.22 (− 0.43, − 0.02)	0.02 (− 0.05, 0.10)	− 0.05 (− 0.09, − 0.01)	− 0.05 (− 0.07, − 0.03)	− 0.02 (− 0.07, 0.03)
Mean difference (95% CI)		− 0.10 (− 1.74, 1.53)	0.13 (− 0.38, 0.64)	− 0.17 (− 0.48, − 0.13)	0.08 (− 0.04, 0.19)	− 0.00 (− 0.06, 0.06)	Ref.	0.03 (− 0.04, 0.10)
220 months (18 years)	14,261							
Mean (95% CI)		0.07 (− 1.20, 1.34)	− 0.06 (− 0.47, 0.35)	− 0.17 (− 0.41, 0.07)	0.03 (− 0.06, 0.13)	− 0.06 (− 0.10, − 0.01)	− 0.08 (− 0.10, − 0.06)	− 0.11 (− 0.17, − 0.05)
Mean difference (95% CI)		0.15 (− 1.76, 2.06)	0.02 (− 0.60, 0.64)	− 0.09 (− 0.45, 0.27)	0.11 (− 0.03, 0.26)	0.02 (− 0.05, 0.09)	Ref.	− 0.03 (− 0.10, 0.12)
Girls								
Birth	30,842							
Mean (95% CI)		− 5.04 (− 5.67, − 4.41)	− 4.00 (− 4.24, − 3.76)	− 3.01 (− 3.18, − 2.84)	− 1.62 (− 1.68, − 1.56)	− 0.56 (− 0.59, − 0.53)	0.10 (0.08, 0.12)	0.43 (0.40, 0.47)
Mean difference (95% CI)		− 5.14 (− 6.09, − 4.19)	− 4.10 (− 4.46, − 3.73)	− 3.11 (− 3.37, − 2.85)	− 1.72 (− 1.81, − 1.63)	− 0.66 (− 0.70, − 0.62)	−	0.33 (0.28, 0.39)
5 months	24,236							
Mean (95% CI)		− 1.09 (− 1.88, − 0.30)	− 0.64 (− 0.94, − 0.35)	− 0.27 (− 0.47, − 0.07)	− 0.10 (− 0.16, − 0.03)	− 0.07 (− 0.10, − 0.04)	− 0.06 (− 0.07, − 0.03)	− 0.04 (− 0.08, − 0.00)
Mean difference (95% CI)		− 1.03 (− 2.22, 0.16)	− 0.59 (− 1.03, − 0.15)	− 0.22 (− 0.52, 0.08)	− 0.04 (− 0.15, 0.06)	− 0.02 (− 0.07, 0.03)	Ref.	0.01 (− 0.05, 0.07)
12 months	23,001							
Mean (95% CI)		− 0.81 (− 1.55, − 0.07)	− 0.70 (− 0.98, − 0.42)	− 0.24 (− 0.44, − 0.04)	− 0.06 (− 0.12, − 0.01)	− 0.06 (− 0.09, − 0.03)	− 0.06 (− 0.08, − 0.04)	− 0.05 (− 0.09, 0.01)
Mean difference (95% CI)		− 0.76 (− 1.87, 0.36)	− 0.64 (− 1.07, − 0.22)	− 0.18 (− 0.48, 0.11)	− 0.00 (− 0.10, 0.10)	− 0.00 (− 0.05, 0.05)	Ref.	0.01 (− 0.05, 0.07)
85 months (7 years)	20,445							
Mean (95% CI)		− 0.73 (− 1.63, 1.17)	− 0.32 (− 0.62, − 0.03)	− 0.14 (− 0.36, − 0.07)	− 0.13 (− 0.21, − 0.05)	− 0.05 (− 0.09, − 0.02)	− 0.05 (− 0.07, − 0.04)	− 0.03 (− 0.08, 0.01)
Mean difference (95% CI)		− 0.68 (− 2.04, 0.68)	− 0.27 (− 0.71, 0.18)	− 0.09 (− 0.41, 0.23)	− 0.07 (− 0.19, 0.04)	− 0.00 (− 0.05, 0.06)	Ref.	0.02 (− 0.04, 0.09)
134 months (11 years)	18,518							
Mean (95% CI)		− 0.47 (− 1.48, 0.54)	− 0.11 (− 0.47, 0.24)	− 0.12 (− 0.35, 0.16)	− 0.04 (− 0.12, 0.04)	− 0.02 (− 0.05, 0.02)	− 0.05 (− 0.07, − 0.03)	− 0.03 (− 0.08, 0.01)
Mean difference (95% CI)		− 0.42 (− 1.94, 1.10)	− 0.07 (− 0.60, 0.47)	− 0.07 (− 0.41, 0.27)	− 0.01 (− 0.11, 0.13)	0.03 (− 0.03, 0.09)	Ref.	0.01 (− 0.06, 0.09)
220 months (18 years)	19,256							
Mean (95% CI)		− 0.36 (− 1.15, 0.44)	− 0.16 (− 0.49, 0.18)	0.02 (− 0.23, 0.26)	− 0.01 (− 0.09, 0.07)	− 0.05 (− 0.09, − 0.01)	− 0.08 (− 0.10, − 0.05)	− 0.04 (− 0.09, 0.01)
Mean difference (95% CI)		− 0.28 (− 1.49, 0.92)	− 0.08 (− 0.59, 0.43)	0.09 (− 0.27, 0.46)	0.06 (− 0.06, 0.19)	0.02 (− 0.04, 0.08)	Ref.	0.03 (− 0.05, 0.11)

Predicted standardized mean birth length and height are weighted to the eligible sample, accounts for clustering of children with same mothers, and adjusted for the mean in continuous variables and the reference categories in categorical variables: maternal pre-pregnancy BMI (23.5), maternal age at delivery (31.2), maternal education (high), maternal height (1.69), equalized household income (4th quartile), maternal smoking during pregnancy (no), gestational diabetes (no), gestational hypertension (no), preeclampsia (no). The mean does not equal zero (at each age), because the estimates are predicted, and adjusted for covariates, clustering of children with same mothers, and inverse probability weights.

By age 7 preterm children had a slightly lower BMI z-score than term children with largest mean differences for very preterm boys (− 0.37, 95% CI: − 0.80 to 0.06) and extremely preterm girls (− 0.68, 95% CI: − 2.04 to 0.68). During adolescence the mean BMI z-score was similar across categories of GA, with the largest mean differences relative to term children observed for extremely preterm girls at both 11 years (− 0.42, 95% CI: − 1.94 to 1.10) and 18 years (− 0.28, 95% CI: − 1.49 to 0.92). Results from the sensitivity analyses with sociodemographic variables were only slightly different with estimates away from the null, while sensitivity analyses without IPW and clustering, respectively, were consistent with the main findings (S3, S4 Tables).

### Trajectories of predicted height z-score by categories of GA

Extremely, very, moderately and late preterm children experienced catch-up in height (gain in z-score of > 0.67) within the first year of life (Table 3, S2 Fig.). By age 1 year, preterm children remained shorter than term children with the largest mean differences observed in extremely preterm boys (− 1.22, 95% CI: − 2.41 to 0.03) and girls (− 1.56, 95% CI: − 2.58 to 0.54) relative to term counterparts.

**Table 3:** Predicted mean height z-scores from birth to age 18 years by category of gestational age, *n* = 62,625 (estimated from LME-model).

Age	Standardized mean height z-score by category of gestational age, and mean differences in height z-score compared to full term reported with 95% Confidence Interval (CI)							
	N	Extremely preterm 23–27 weeks	Very preterm 28–31 weeks	Moderately preterm 32–33 weeks	Late preterm 34–36 weeks	Early term 37–38 weeks	Term 39–41 weeks	Post term 42–43 weeks
Boys								
Birth	31,407							
Mean (95% CI)		− 6.40 (− 7.10, − 5.72)	− 4.31 (− 4.53, − 4.09)	− 2.83 (− 2.97, − 2.68)	− 1.39 (− 1.44, − 1.33)	− 0.44 (− 0.47, − 0.41)	0.17 (0.15, 0.19)	0.55 (0.52, 0.59)
Mean difference (95% CI)		− 6.56 (− 7.63, − 5.50)	− 4.48 (− 4.81, − 4.15)	− 2.99 (− 3.21, − 2.78)	− 1.56 (− 1.64, − 1.48)	− 0.62 (− 0.65, − 0.57)	Ref.	0.38 (0.33, 0.43)
5 months	25,050							
Mean (95% CI)		− 2.73 (− 3.89, − 1.57)	− 2.37 (− 2.63, − 2.11)	− 1.58 (− 1.74, − 1.42)	− 0.67 (− 0.73, − 0.61)	− 0.22 (− 0.25, − 0.19)	0.10 (0.08, 0.12)	0.30 (0.26, 0.34)
Mean difference (95% CI)		− 2.83 (− 4.58, − 1.08)	− 2.47 (− 2.86, − 2.08)	− 1.68 (− 1.92, − 1.43)	− 0.77 (− 0.86, − 0.68)	− 0.32 (− 0.36, − 0.27)	Ref.	0.20 (0.14, 0.26)
12 months	23,714							
Mean (95% CI)		− 1.18 (− 1.97, − 0.39)	− 0.81 (− 1.07, − 0.56)	− 0.56 (− 0.73, − 0.40)	− 0.15 (− 0.21, − 0.09)	− 0.06 (− 0.09, − 0.03)	0.04 (0.02, 0.06)	0.16 (0.13, 0.20)
Mean difference (95% CI)		− 1.22 (− 2.41, − 0.03)	− 0.85 (− 1.23, − 0.48)	− 0.60 (− 0.85, − 0.36)	− 0.19 (− 0.28, − 0.10)	− 0.10 (− 0.14, − 0.05)	Ref.	0.13 (0.07, 0.18)
85 months (7 years)	21,774							
Mean (95% CI)		− 0.80 (− 1.58, − 0.02)	− 0.14 (− 0.40, 0.11)	− 0.28 (− 0.45, − 0.11)	− 0.05 (− 0.11, − 0.02)	0.00 (− 0.03, 0.04)	0.02 (0.00, 0.04)	0.07 (0.03, 0.11)
Mean difference (95% CI)		− 0.82 (− 1.99, 0.35)	− 0.17 (− 0.55, 0.22)	− 0.30 (− 0.56, − 0.05)	− 0.07 (− 0.17, 0.03)	− 0.02 (− 0.07, 0.03)	Ref.	0.05 (− 0.01, 0.11)
134 months (11 years)	18,555							
Mean (95% CI)		− 0.65 (− 1.64, 0.33)	− 0.11 (− 0.41, 0.19)	− 0.26 (− 0.44, − 0.07)	− 0.01 (− 0.08, 0.06)	0.03 (− 0.00, 0.07)	0.02 (− 0.00, 0.03)	0.03 (− 0.02, 0.07)
Mean difference (95% CI)		− 0.67 (− 2.15, 0.81)	− 0.13 (− 0.59, 0.33)	− 0.27 (− 0.55, 0.01)	− 0.02 (− 0.13, 0.08)	0.02 (− 0.04, 0.07)	Ref.	0.01 (− 0.06, 0.08)
220 months (18 years)	14,657							
Mean (95% CI)		− 0.53 (− 1.67, 0.62)	− 0.35 (− 0.71, 0.01)	− 0.13 (− 0.35, 0.09)	− 0.07 (− 0.16, 0.02)	0.04 (-0.00, 0.08)	0.03 (0.01, 0.06)	0.05 (− 0.01, 0.10)
Mean difference (95% CI)		− 0.56 (− 2.28, 1.16)	− 0.39 (− 0.93, 0.16)	− 0.16 (− 0.50, 0.17)	− 0.10 (− 0.24, 0.03)	0.00 (− 0.06, 0.07)	Ref.	0.01 (− 0.07, 0.09)
Girls								
Birth	31,218							
Mean (95% CI)		− 6.69 (− 7.25, − 6.13)	− 4.64 (− 4.87, − 4.42)	− 2.91 (− 3.07, − 2.75)	− 1.42 (− 1.48, − 1.36)	− 0.42 (− 0.45, − 0.39)	0.18 (0.16, 0.20)	0.54 (0.50, 0.57)
Mean difference (95% CI)		− 6.87 (− 7.72, − 6.03)	− 4.82 (− 5.16, − 4.49)	− 3.09 (− 3.33, − 2.85)	− 1.60 (− 1.69, − 1.52)	− 0.60 (− 0.64, − 0.56)	Ref.	0.36 (0.31, 0.42)
5 months	24,341							
Mean (95% CI)		− 2.63 (− 3.36, − 1.90)	− 2.16 (− 2.43, − 1.90)	− 1.46 (− 1.65, − 1.28)	− 0.67 (− 0.73, − 0.60)	− 0.19 (− 0.22, − 0.16)	0.10 (0.08, 0.12)	0.29 (0.25, 0.33)
Mean difference (95% CI)		− 2.73 (− 3.83, − 1.63)	− 2.26 (− 2.67, − 1.86)	− 1.57 (− 1.85, − 1.29)	− 0.77 (− 0.86, − 0.67)	− 0.30 (− 0.34, − 0.25)	Ref.	0.18 (0.12, 0.24)
12 months	23,079							
Mean (95% CI)		− 1.50 (− 2.17, − 0.82)	− 1.07 (− 1.33, − 0.81)	− 0.70 (− 0.88, − 0.51)	− 0.24 (− 0.31, − 0.18)	− 0.06 (− 0.10, − 0.03)	0.06 (0.04, 0.08)	0.18 (0.14, 0.22)
Mean difference (95% CI)		− 1.56 (− 2.58, 0.54)	− 1.13 (− 1.52, − 0.74)	− 0.76 (− 1.04, − 0.49)	− 0.31 (− 0.40, − 0.21)	− 0.13 (− 0.17, − 0.08)	Ref.	0.12 (0.06, 0.18)
85 months (7 years)	21,033							
Mean (95% CI)		− 0.86 (− 1.68, − 0.04)	− 0.29 (− 0.55, − 0.02)	− 0.29 (− 0.49, − 0.10)	− 0.06 (− 0.13, 0.01)	0.03 (− 0.01, 0.06)	0.04 (0.02, 0.06)	0.07 (0.03, 0.11)
Mean difference (95% CI)		− 0.90 (− 2.14, 0.34)	− 0.33 (− 0.72, 0.07)	− 0.33 (− 0.63, − 0.04)	− 0.10 (− 0.20, 0.01)	− 0.01 (− 0.07, 0.04)	Ref.	0.03 (− 0.03, 0.09)
134 months (11 years)	19,011							
Mean (95% CI)		− 0.52 (− 1.41, 0.36)	− 0.07 (− 0.39, 0.25)	− 0.20 (− 0.40, 0.02)	− 0.08 (− 0.15, − 0.01)	0.02 (− 0.02, 0.06)	0.04 (0.02, 0.06)	0.06 (0.02, 0.09)
Mean difference (95% CI)		− 0.56 (− 1.90, 0.77)	− 0.11 (− 0.59, 0.38)	− 0.23 (− 0.55, 0.08)	− 0.12 (− 0.23, − 0.00)	− 0.01 (− 0.07, 0.04)	Ref.	0.02 (− 0.05, 0.09)
220 months (18 years)	20,533							
Mean (95% CI)		− 0.93 (− 1.65, − 0.22)	− 0.22 (− 0.53, 0.09)	− 0.29 (− 0.51, − 0.06)	− 0.04 (− 0.12, 0.04)	0.04 (0.00, 0.08)	0.06 (0.04, 0.08)	0.02 (− 0.03, 0.07)
Mean difference (95% CI)		− 1.00 (− 2.08, 0.08)	− 0.28 (− 0.75, 0.18)	− 0.35 (− 0.69, − 0.01)	− 0.10 (− 0.22, 0.01)	− 0.02 (− 0.08, 0.04)	Ref.	− 0.04 (− 0.12, 0.03)

Predicted standardized mean birth length and height are weighted to the eligible sample, accounts for clustering of children with same mothers, and adjusted for the mean in continuous variables and the reference categories in categorical variables: maternal pre-pregnancy BMI (23.5), maternal age at delivery (31.2), maternal education (high), maternal height (1.69), equalized household income (4th quartile), maternal smoking during pregnancy (no), gestational diabetes (no), gestational hypertension (no), and preeclampsia (no). The mean does not equal zero (at each age), because the estimates are predicted, and adjusted for covariates, clustering of children with same mothers, and inverse probability weights.

The mean difference in height z-score between preterm and term attenuated between age 1 year and 7 years, yet extremely, very and moderately preterm remained relatively shorter than term. By age 11 and through age 18 years individuals born preterm remained slightly shorter than term with mean differences being greatest in extremely preterm girls (− 1.00, 95% CI: − 2.08 to 0.08). Results from the sensitivity analyses were overall similar to the main finding, though the analysis with adjustment for sociodemographic variables only scarcely changed the estimates away from the null (S5, S6 Tables).

## Discussion

This study investigated trajectories of predicted BMI and height z-scores across categories of GA from birth through 18 years. In the first year of life, BMI and height were lower in preterm than in term infants. Mean difference in BMI and height between preterm and term attenuated during childhood, and continued to decrease towards zero for BMI by adolescence. At 18 years of age, individuals born preterm remained only slightly shorter than children born at term.

This study agrees with two recent publications from United Kingdom (UK) (*n* = 475) and Australia (*n* = 478) showing that a mean difference in BMI between extremely preterm and term is largest in infancy, whilst decreasing during childhood through adolescence^21,45^. The two studies reported lower mean difference in BMI at age 6 years (− 0.98, 95% CI: − 1.23 to − 0.73) and 8 years (− 0.42, 95% CI: − 0.67 to − 0.18), respectively, which corresponds the magnitude of association for our results in girls at age 7 years (− 0.71, 95% CI: − 2.10 to 0.67). Similar to our findings, the studies from UK and Australia found no difference in BMI for extremely preterm and term adolescents at age 18 and 19 years, respectively.

Very and moderately preterm boys and girls remained lighter than term after birth through 7 years in our study. This is in line with findings from a recent cohort study from Brazil (*n* = 3036) reporting lower mean BMI in both boys and girls (born ≤ 33 weeks) aged 6 years^25^. Also, we found that late preterm had similar mean BMI as term already following the first 5 months of life through adolescence, as reported previously in a Chinese study on 7 169 children aged 14 years, and at age 18 years in the study from Brazil^46^.

The studies from Australia, UK, Brazil and China did not adjust for potential confounders such as maternal pre-pregnancy BMI and pre-eclampsia. While the results are in accordance with our findings, we consider it a strength of our study that the estimates are adjusted for key confounders and sex-specific across seven categories of GA contrasting previous publications.

We further found evidence to support that GA is positively associated with height in infancy with results indicating a linear association. This is in line with findings from a British study on 18 818 singletons aged 3 and 5 years, despite the authors reporting greater magnitudes of association for each GA-category (23–31 weeks, 32–33 weeks, 34–36 weeks, 37–38 weeks), respectively^3^. The analyses, however, were not adjusted for maternal height, gestational diabetes, gestational hypertension or preeclampsia.

For extremely preterm children, our study suggests that the height remain shorter after birth through adolescence, although the mean differences attenuate with age. These findings agree with results from Australia and UK where extremely preterm was shorter than term peers through infancy, childhood and adolescence compared to term peers^21,45^. The study from UK reported a lower mean height z-score in 315 extremely preterm aged 6 years (− 0.95, 95% CI: − 1.16 to − 0.73), 11 years (− 0.71, 95% CI: − 0.92 to − 0.50), and 19 years (− 0.81, 95% CI: − 1.14 to − 0.47), respectively, compared with term peers, which corresponds to the likes of the study from Australia.

Our findings support that gestational duration affects height in early life, and that this pattern persists and includes the age where individuals reach their maximum height. This contrast previous genetically informed studies challenging that exposures during fetal life is associated with postnatal growth^47^.

A strength of this study is the large sample size, and the ability to assess associations of seven categories of GA with BMI and height at birth and at scheduled ages 5 months, 1, 7, 11 and 18 years. Also, we were able to include maternal anthropometrics and measures of gestational hypertension and diabetes and preeclampsia.

A key limitation of our study is missing data on one-third of participants due to loss-to-follow up or lack of reporting child height and weight, or maternal pre-pregnancy BMI. To account for potential biases due to missing data, in the main analyses we accounted for missing data using IPWs and found that results were broadly consistent with results obtained in sensitivity analyses without IPWs (S3–S6 Tables).

Lack of growth measurements between age 2 to 5 and 13 to 16 years is also a limitation, which means we are not able to make any inference during two periods of dynamic change in size, namely adiposity rebound and puberty.

We relied on parental reporting of child weight and height from 7 to 11 years, and self-report at 18 years. Parental reporting at child height and weight may be prone to systematic error of under- or over-reporting of BMI, but given characteristics of the data collections we do not assume this would affect our overall estimates markedly^48^. Self-reporting of current BMI is a reasonably valid tool^49^, hence we neither considered this to bias the results. Our study uses data from a healthy population^50^ and there is a slightly overrepresentation of socially advantaged families in the study population (S1 Table). However, given the particular magnitudes we do not consider this to affect the validity of internal comparisons within the cohort, while earlier studies have also demonstrated that estimates obtained from the DNBC have been almost identical to those from completely unselected populations^50^. Also, the sensitivity analyses without IPW were consistent with the main analyses. DNBC is almost entirely comprised of women of Danish ethnic origin, thus replication of our findings in more diverse populations might be useful.

Importantly, to understand the causal mechanisms between the associations of GA and BMI and height, respectively, intermediate factors such as birth weight and breast feeding would be relevant to include for investigate in future studies.

## Clinical implications

For preterm infants, the largest mean differences in BMI and height relative to term infants appear in the first years of life. However, despite magnitudes of potential clinical relevance in infancy (mean difference > 0.5 z-score)^51^, findings from our main and sensitivity analyses are reassuring for the growth of preterm individuals with the majority of children reaching similar height and BMI of term peers by end of adolescence Importantly, for a clinical setting these findings should be further considered in combination with maternal characteristics (e.g., socioeconomic position, gestational diabetes and hypertension, preeclampsia, smoking during pregnancy, and comorbidities).

## Conclusion

The lower BMI in preterm infants relative to term infants equalizes during childhood, such that by adolescence there is no clear difference. Height is strongly positively associated with GA in early childhood, whilst by end of adolescence preterm individuals remain only slightly shorter than term peers.

## Supplementary Information

Supplementary Information.

## Data Availability

All data were accessed and analysed using remote access to Statistics Denmark, where data were anonymized and the non-visibility of individual data secured. Availability of these data is restricted to research institutions with approved license such as Danish public universities and are not publicly available. Access to data can be given upon reasonable request and with permission from Statistics Denmark.
