# Supplementary material for: Gestational age and trajectories of body mass index and height from birth through adolescence in the Danish National Birth Cohort
Source: Sci Rep. 2023 Feb 26;13:3298. doi: 10.1038/s41598-023-30123-y (PMC9968714; doi:10.1038/s41598-023-30123-y)
Supplement: Supplementary file 1 — Supplementary Information. [file 41598_2023_30123_MOESM1_ESM.docx]

**Gestational age and trajectories of body mass index and height from birth through adolescence in the Danish National Birth Cohort**

Johan L. Vinther*, MSc; Claus T. Ekstrøm, PhD; Thorkild I.A. Sørensen, Dr Med Sci; Luise Cederkvist PhD; Deborah A. Lawlor, PhD; Anne-Marie Nybo Andersen, PhD

**Supplementary Material**

**Supplementary Table S1.** Baseline characteristics comparing analytic and excluded sample

**Supplementary Table S2.** Descriptive measurements of height and BMI in analysis sample by category of GA

**Supplementary Table S3.** Mean BMI z-score in boys from birth to 18 years, by category of GA

**Supplementary Table S4.** Mean BMI z-score in girls birth to 18 years, by category of GA

**Supplementary Table S5.** Mean height z-score in boys birth to 18 years, by category of GA

**Supplementary Table S6.** Mean height z-score in girls birth to 18 years, by category of GA

**Supplementary Figure S1.** Sex-specific BMI z-score trajectory from birth to 18 years, by category of GA

**Supplementary Figure S2.** Sex-specific height z-score trajectory from birth to 18 years, by category of GA

| **Supplementary Table S1.** Baseline characteristics comparing analysis and excluded sample, *n = 92 615* | | |
| --- | --- | --- |
|  | **Analysis Sample** | Excluded Sample |
| Total N of Children | **62 625** | 29 990 |
| Sex, *female* (%) | **49.8** | 46.5 |
| Gestational Age, mean (SD), days | **280 (11)** | 279 (15) |
| Birth Weight, mean (SD), g | **3 602 (546)** | 3 531 (652) |
| Maternal Height (m) | **1.69** | 1.69 |
| Maternal Age at Delivery, mean, years | **31.1** | 30.5 |
| Maternal Age at Delivery (%) |  |  |
| <25 | **4.7** | 9.3 |
| 25-30 | **32.1** | 33.2 |
| 31-35 | **41.9** | 38.6 |
| >35 | **21.3** | 18.8 |
| Maternal Smoking during Pregnancy (%) | **24.4** | 28.2 |
| Maternal pre-pregnancy BMI, mean (SD), kg/m^2^ | **23.6 (4.2)** | 23.6 (4.4) |
| Maternal pre-pregnancy BMI, (%) |  |  |
| <18.5 | **4.2** | 5.4 |
| 18.5-25.0 | **68.4** | 66.2 |
| >25.0 | **27.4** | 28.4 |
| Level of Highest or ongoing Education (ISCED/11) (%) |  |  |
| Low | **9.1** | 16.7 |
| Medium | **42.5** | 42.9 |
| High | **48.4** | 40.4 |
| Equivalised Income, Quartiles (%) |  |  |
| 1 (Lowest) | **22.0** | 31.4 |
| 2 | **25.5** | 23.8 |
| 3 | **26.2** | 22.5 |
| 4 (Highest) | **26.3** | 22.3 |
| Gestational Diabetes, *Yes* (%) | **0.8** | 0.8 |
| Gestational Hypertension, *Yes* (%) | **4.9** | 5.3 |
| Preeclampsia, *Yes* (%) | **2.3** | 2.8 |
| Mode of Delivery, *Caesarean Section* (%) | **14.8** | 16.7 |
| BMI at 5 months data collection, mean (SD), kg/m^2^ | **16.7 (1.6)** | 16.7 (1.8) |
| BMI at 12 months data collection, mean (SD), kg/m^2^ | **17.0 (1.6)** | 17.1 (1.7) |
| BMI at 7 years data collection, mean (SD), kg/m^2^ | **15.7 (1.7)** | 15.8 (1.9) |
| BMI at 11 years data collection, mean (SD), kg/m^2^ | **17.4 (2.5)** | 17.7 (2.6) |
| BMI at 18 years data collection, mean (SD), kg/m^2^ | **22.3 (3.9)** | 22.7 (4.2) |

* SD = Standard Deviation, BMI = Body Mass Index, ISCED = International Standard Classification of Education

| **Supplementary Table S2.** Descriptive measurements of height and BMI in analysis sample by category of gestational age (n = 61 969 – 62 625) | | | | | | | | |
| --- | --- | --- | --- | --- | --- | --- | --- | --- |
|  | **Extremely**  **Preterm  23-27 weeks** | **Very  Preterm  28-31 weeks** | **Moderately Preterm  32-33 weeks** | **Late  Preterm  34-36 weeks** | **Early**  **Term  37-38 weeks** | **Term  39-41 weeks** | **Post  Term  42-43 weeks** | **Total  23-43 weeks** |
| Age at 5 months data collection, mean (SD), months | 5.8 (1.6) | 5.3 (1.0) | 5.2 (0.8) | 5.0 (0.6) | 5.0 (0.6) | 5.0 (0.6) | 5.0 (0.6) | **5.0 (0.6)** |
| Age at 12 months data collection, mean (SD), months | 12.6 (1.1) | 12.4 (1.0) | 12.3 (0.9) | 12.2 (0.9) | 12.2 (0.9) | 12.2 (0.9) | 12.2 (0.9) | **12.2 (0.9)** |
| Age at 7 years data collection, mean (SD), months | 86.0 (2.4) | 85.1 (4.2) | 84.9 (4.0) | 85.3 (3.6) | 85.2 (3.6) | 85.2 (3.6) | 85.1 (3.7) | **85.2 (3.6)** |
| Age at 11 years data collection, mean (SD), months | 140.7 (9.4) | 136.4 (6.2) | 136.4 (6.0) | 137.6 (7.0) | 137.5 (6.8) | 137.7 (6.9) | 137.8 (7.0) | **137.7 (6.9)** |
| Age at 18 years data collection, mean (SD), months | 216.0 (6.1) | 217.2 (5.3) | 217.9 (5.2) | 217.7 (5.2) | 217.6 (5.2) | 217.7 (5.1) | 217.8 (5.1) | **217.7 (5.1)** |
| Length at birth, mean (SD), cm | 36.2 (3.6) | 41.0 (3.5) | 45.0 (3.0) | 48.7 (2.6) | 51.1 (2.2) | 52.7 (2.1) | 53.7 (2.1) | **52.3 (2.5)** |
| Height at 5 months data collection (SD), cm | 60.9 (5.0) | 62.2 (3.7) | 64.2 (3.5) | 66.3 (3.0) | 67.6 (2.8) | 68.4 (2.8) | 69.1 (2.8) | **68.2 (2.9)** |
| Height (cm) at 12 months data collection, mean (SD), cm | 73.3 (3.4) | 74.6 (3.6) | 75.5 (3.6) | 76.8 (3.2) | 77.3 (3.1) | 77.7 (3.1) | 78.1 (3.1) | **77.6 (3.1)** |
| Height (cm) at 7 years data collection, mean (SD), cm | 121.3 (5.6) | 123.8 (5.6) | 123.7 (6.5) | 125.2 (5.8) | 125.5 (5.6) | 125.7 (5.6) | 160.1 (5.5) | **125.7 (5.6)** |
| Height (cm) at 11 years data collection, mean (SD), cm | 146.3 (7.8) | 148.4 (6.3) | 148.0 (8.5) | 150.1 (8.1) | 150.7 (7.9) | 151.0 (7.9) | 151.4 (7.9) | **150.9 (7.9)** |
| Height (cm) at 18 years data collection, mean (SD), cm | 168.3 (8.4) | 173.1 (9.6) | 174.4 (9.9) | 174.6 (9.7) | 175.5 (9.8) | 175.6 (7.5) | 175.9 (7.4) | **175.2 (9.6)** |
| BMI at birth, mean (SD), kg/m^2^ | 7.2 (1.0) | 8.7 (1.3) | 10.0 (1.3) | 11.5 (1.4) | 12.7 (1.3) | 13.2 (1.2) | 13.5 (1.3) | **13.1 (1.4)** |
| BMI at 5 months data collection, mean (SD), kg/m^2^ | 15.0 (2.1) | 15.5 (1.7) | 16.4 (1.8) | 16.6 (1.7) | 16.7 (1.7) | 16.7 (1.6) | 16.8 (1.6) | **16.7 (1.6)** |
| BMI at 12 months data collection, mean (SD), kg/m^2^ | 15.6 (1.9) | 16.2 (1.8) | 16.9 (1.7) | 17.0 (1.6) | 17.0 (1.6) | 17.0 (1.6) | 17.1 (1.6) | **17.0 (1.6)** |
| BMI at 7 years data collection, mean (SD), kg/m^2^ | 14.9 (1.6) | 15.2 (1.9) | 15.3 (1.6) | 15.6 (1.9) | 15.7 (1.7) | 15.7 (1.7) | 15.8 (1.7) | **15.7 (1.7)** |
| BMI at 11 year data collection, mean (SD), kg/m^2^ | 16.8 (2.7) | 17.5 (2.8) | 16.8 (2.1) | 17.5 (2.5) | 17.4 (2.5) | 17.4 (2.4) | 17.5 (2.5) | **17.4 (2.5)** |
| BMI at 18 year data collection, mean (SD), kg/m^2^ | 21.5 (3.2) | 22.4 (4.5) | 22.0 (3.1) | 22.5 (3.7) | 22.3 (3.9) | 22.3 (3.8) | 22.5 (4.2) | **22.3 (3.9)** |

* BMI = Body Mass Index, GA = Gestational Age at Birth, SD = Standard Deviation,

**Supplementary Figure S1.** Sex-specific predicted mean BMI z-score trajectory by category of gestational age and sex

The figures show the predicted mean BMI trajectories in boys (**a**) and girls (**b**) from birth (0 months) to age 228 months by categories of gestational age. Shaded areas around the mean trajectories represent 95% confidence intervals, and the bottom black lines illustrate where data were available (*n = 61 969)*

(**a**)

**
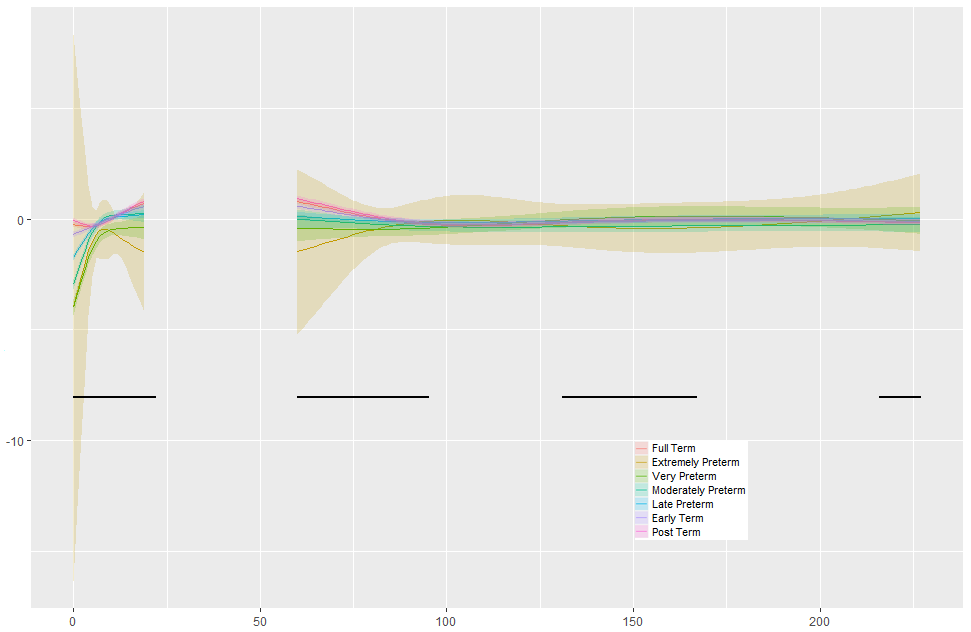

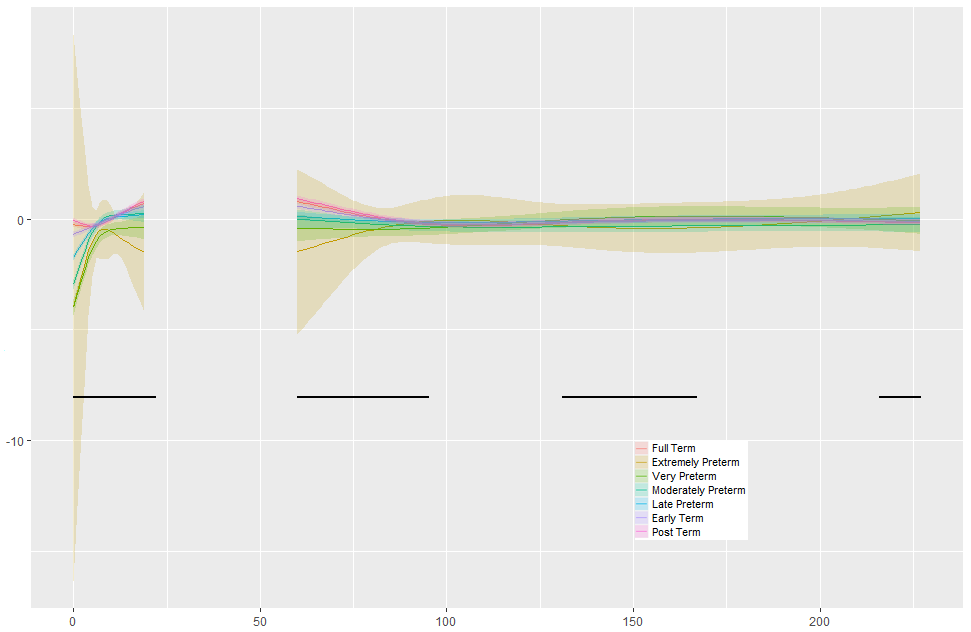
***
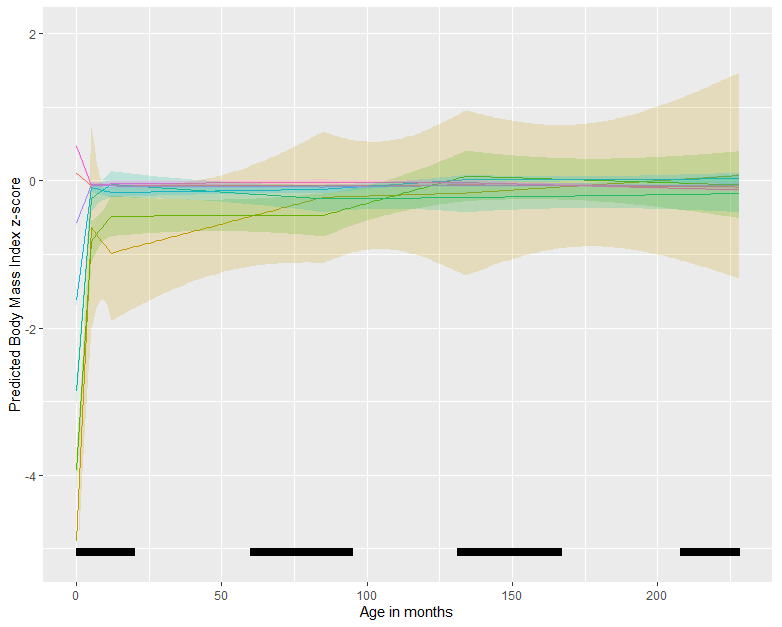
*

(**b**)

**
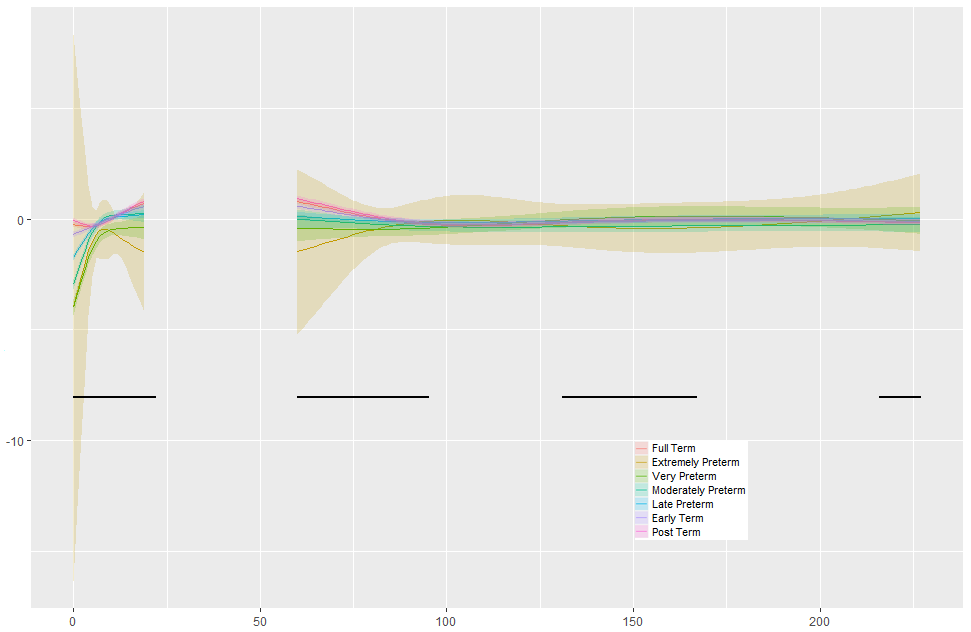

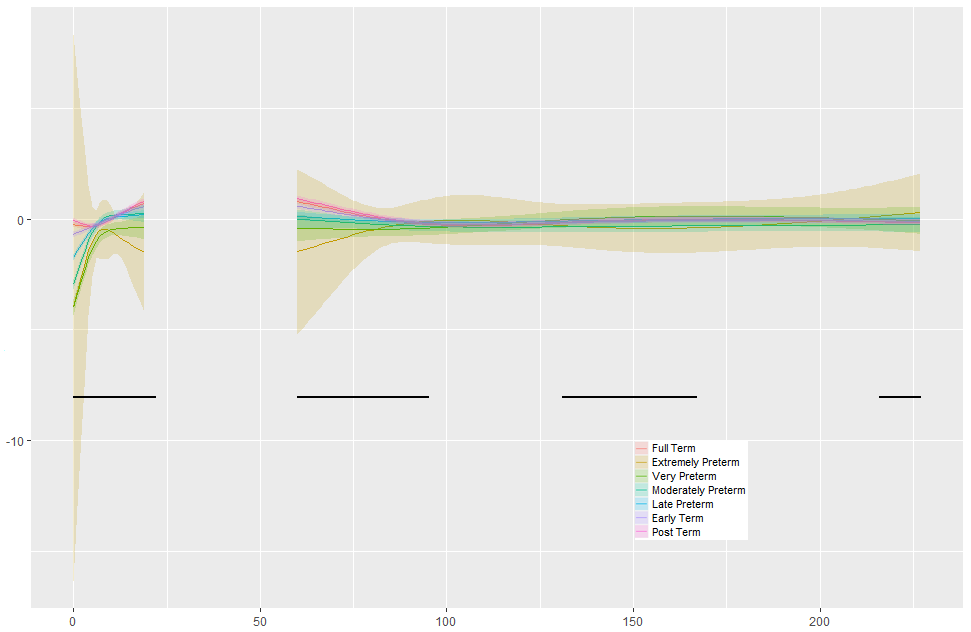
**
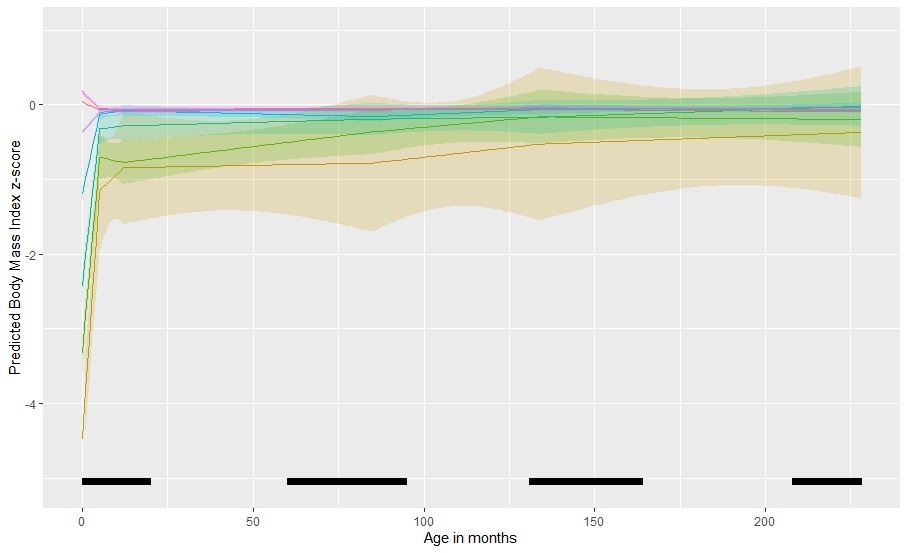


**Supplementary Figure S2.** Sex-specific predicted height z-score trajectory by category of gestational age

The figures show the predicted mean height trajectories in boys (**a**) and girls (**b**) from birth (0 months) to age 228 months by categories of gestational age. Shaded areas around the mean trajectories represent 95% confidence intervals, and the bottom black lines illustrate where data were available (*n = 62 625*)

**
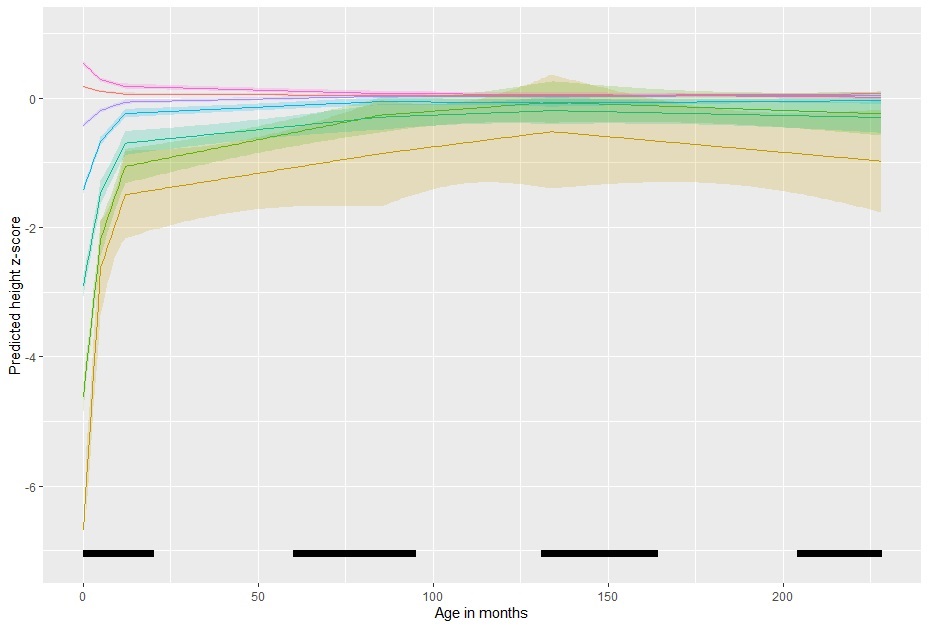
**(**a**)

**
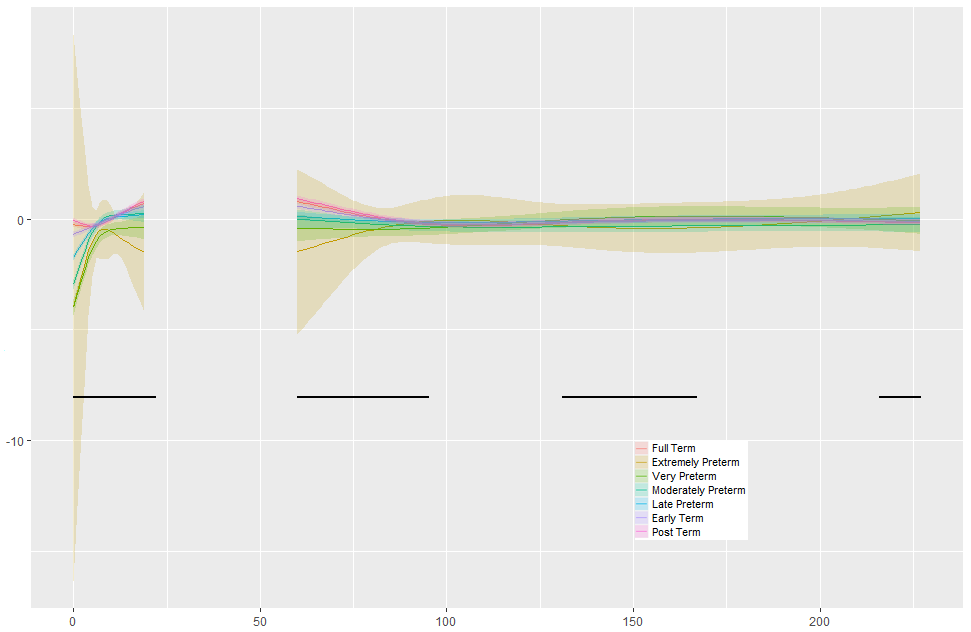

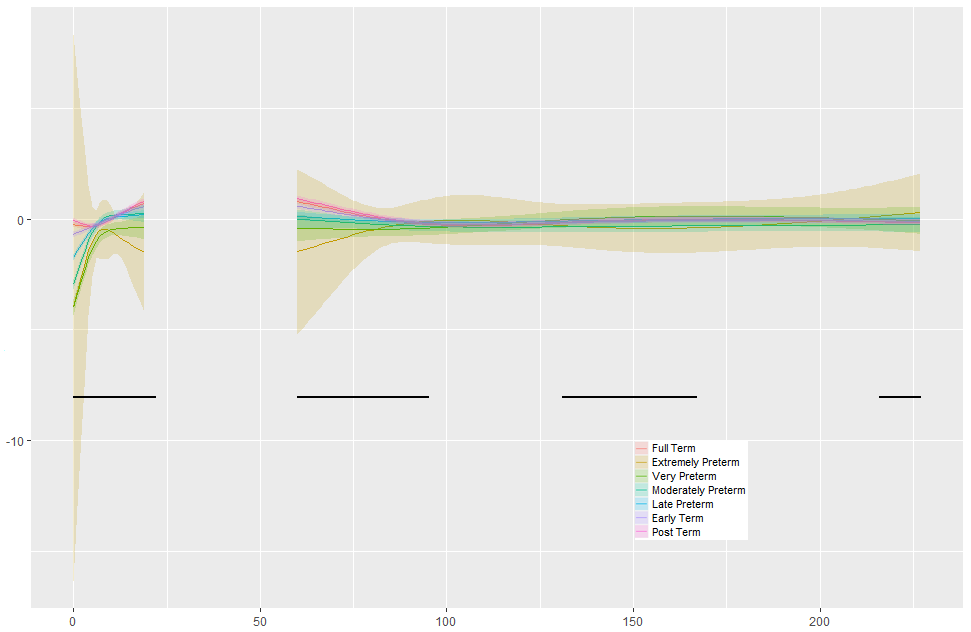
**

(**b**)**
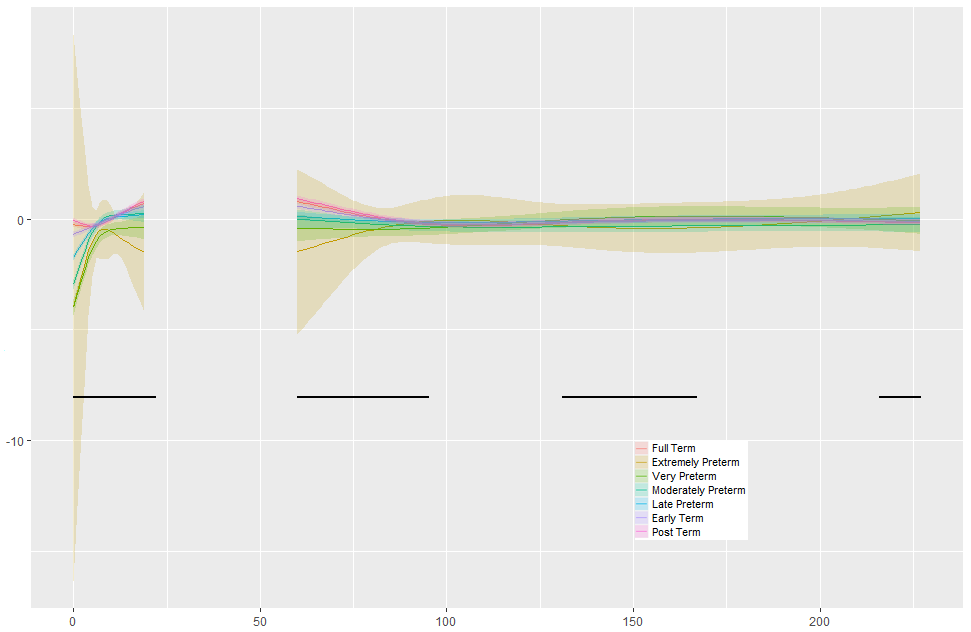
**

**
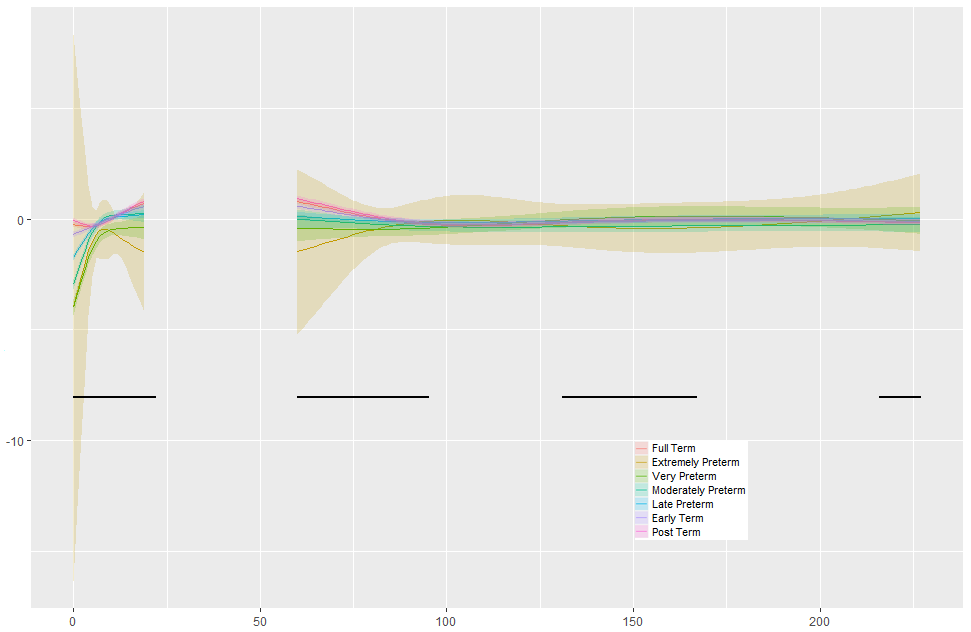

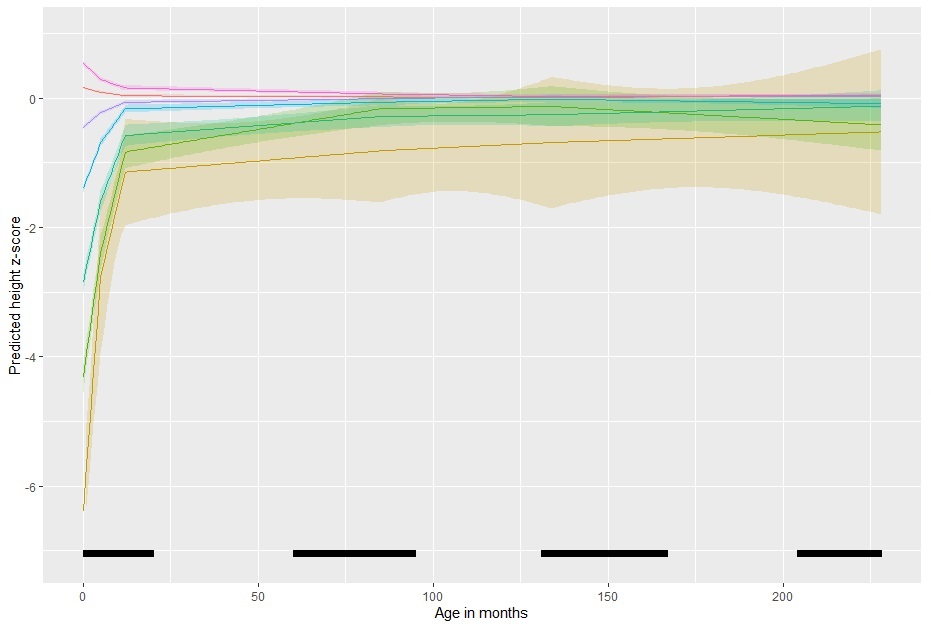
**

| **Supplementary Table S3.** Predicted mean BMI z-score in boys, by category of gestational age (95% CI) (*n = 31 127*) | | | | | | | |  |
| --- | --- | --- | --- | --- | --- | --- | --- | --- |
|  | **Extremely Preterm**  **23-27 weeks** | **Very Preterm**  **28-31 weeks** | **Moderately Preterm**  **32-33 weeks** | **Late Preterm**  **34-36 weeks** | **Early Term**  **37-38 weeks** | **Term**  **39-41 weeks** | **Post Term**  **42-43 weeks** | |
| **Birth** |  |  |  |  |  |  |  | |
| Model 1 | –4.32 (–4.82,–3.83) | –3.25 (–3.46,–3.05) | –2.22 (–2.36,–2.07) | –1.18 (–1.23,–1.12) | –0.31 (–0.34,–0.29) | 0.12 (0.10, 0.13) | 0.31 (0.28, 0.34) | |
| Model 2 | –4.98 (–5.75,–4.20) | –3.93 (–4.18,–3.68) | –2.87 (–3.02,–2.71) | –1.65 (–1.70,–1.59) | –0.59 (–0.62,–0.58) | 0.09 (0.07, 0.11) | 0.49 (0.45, 0.52) | |
| Model 3 | –4.90 (–5.67,–4.12) | –3.92 (–4.16,–3.67) | –2.84 (–2.99,–2.69) | –1.62 (–1.68,–1.56) | –0.57 (–0.59,–0.54) | 0.11 (0.09, 0.12) | 0.48 (0.44, 0.51) | |
| Model 4 | –4.90 (–5.67,–4.12) | –3.92 (–4.16,–3.67) | –2.83 (–2.99,–2.68) | –1.62 (–1.68,–1.56) | –0.56 (–0.59,–0.53) | 0.11 (0.09, 0.13) | 0.48 (0.44, 0.52) | |
| Model 5 | –4.88 (–5.37,–4.40) | –3.91 (–4.11,–3.70) | –2.86 (–3.00,–2.72) | –1.63 (–1.69,–1.57) | –0.57 (–0.60,–0.54) | 0.10 (0.08, 0.12) | 0.47 (0.44, 0.51) | |
|  |  |  |  |  |  |  |  | |
| **5 months** |  |  |  |  |  |  |  | |
| Model 1 | –0.36 (–1.17, 0.46) | –0.78 (–1.02,–0.54) | –0.18 (–0.35,–0.02) | –0.05 (–0.11, 0.02) | 0.01 (–0.02, 0.04) | –0.00 (–0.02, 0.01) | 0.03 (–0.00, 0.07) | |
| Model 2 | –0.50 (–1.83, 0.83) | –0.82 (–1.11,–0.53) | –0.22 (–0.40,–0.05) | –0.08 (–0.15,–0.02) | –0.04 (–0.08,–0.01) | –0.06 (–0.08,–0.04) | –0.06 (–0.10,–0.02) | |
| Model 3 | –0.47 (–1.80, 0.86) | –0.80 (–1.09,–0.52) | –0.22 (–0.40,–0.05) | –0.08 (–0.15,–0.02) | –0.04 (–0.08,–0.01) | –0.06 (–0.08,–0.04) | –0.06 (–0.10,–0.02) | |
| Model 4 | –0.46 (–1.80, 0.87) | –0.81 (–1.09,–0.52) | –0.22 (–0.40,–0.04) | –0.08 (–0.15,–0.02) | –0.04 (–0.07,–0.01) | –0.06 (–0.08,–0.04) | –0.05 (–0.09,–0.01) | |
| Model 5 | –0.38 (–1.18, 0.41) | –0.84 (–1.07,–0.60) | –0.23 (–0.39,–0.06) | –0.08 (–0.14,–0.02) | –0.04 (–0.07,–0.01) | –0.06 (–0.08,–0.04) | –0.06 (–0.10,–0.01) | |
|  |  |  |  |  |  |  |  | |
| **1 year** |  |  |  |  |  |  |  | |
| Model 1 | –0.74 (–1.31,–0.17) | –0.42 (–0.66,–0.19) | 0.01 (–0.16, 0.17) | –0.11 (–0.17,–0.04) | –0.01 (–0.04, 0.02) | –0.00 (–0.02, 0.01) | 0.05 (0.01, 0.09) | |
| Model 2 | –0.89 (–1.78, 0.00) | –0.47 (–0.75,–0.20) | –0.06 (–0.24, 0.12) | –0.18 (–0.24,–0.11) | –0.08 (–0.12,–0.05) | –0.08 (–0.10,–0.06) | –0.03 (–0.07, 0.01) | |
| Model 3 | –0.84 (–1.73, 0.04) | –0.46 (–0.74,–0.19) | –0.04 (–0.21, 0.14) | –0.15 (–0.22,–0.09) | –0.06 (–0.09,–0.03) | –0.06 (–0.08,–0.04) | –0.03 (–0.07, 0.01) | |
| Model 4 | –0.84 (–1.73, 0.05) | –0.46 (–0.74,–0.19) | –0.03 (–0.20, 0.15) | –0.15 (–0.22,–0.09) | –0.06 (–0.09,–0.03) | –0.06 (–0.08,–0.04) | –0.03 (–0.07, 0.01) | |
| Model 5 | –0.76 (–1.32,–0.21) | –0.49 (–0.72,–0.27) | –0.04 (–0.20, 0.12) | –0.15 (–0.22,–0.09) | –0.06 (–0.09,–0.03) | –0.06 (–0.08,–0.04) | –0.03 (–0.07, 0.01) | |
|  |  |  |  |  |  |  |  | |
| **7 years** |  |  |  |  |  |  |  | |
| Model 1 | –0.33 (–0.86, 0.19) | –0.30 (–0.54,–0.06) | –0.19 (–0.35,–0.02) | –0.06 (–0.12, 0.01) | –0.02 (–0.05, 0.00) | –0.01 (–0.02,–0.00) | 0.07 (0.04, 0.11) | |
| Model 2 | –0.35 (–1.22, 0.53) | –0.45 (–0.74,–0.16) | –0.28 (–0.46,–0.10) | –0.13 (–0.20,–0.06) | –0.10 (–0.13,–0.06) | –0.09 (–0.11,–0.07) | –0.00 (–0.05, 0.04) | |
| Model 3 | –0.23 (–1.10, 0.63) | –0.44 (–0.73,–0.15) | –0.24 (–0.43,–0.06) | –0.11 (–0.18,–0.04) | –0.08 (–0.11,–0.04) | –0.07 (–0.09,–0.05) | –0.01 (–0.05, 0.03) | |
| Model 4 | –0.23 (–1.10, 0.64) | –0.44 (–0.73,–0.15) | –0.24 (–0.42,–0.06) | –0.11 (–0.17,–0.04) | –0.07 (–0.11,–0.04) | –0.07 (–0.09,–0.05) | –0.01 (–0.05, 0.03) | |
| Model 5 | –0.31 (–0.83, 0.20) | –0.44 (–0.67,–0.20) | –0.23 (–0.40,–0.07) | –0.10 (–0.17,–0.04) | –0.07 (–0.11,–0.04) | –0.06 (–0.08,–0.04) | –0.01 (–0.05, 0.04) | |
|  |  |  |  |  |  |  |  | |
| **11 years** |  |  |  |  |  |  |  | |
| Model 1 | –0.21 (–0.88, 0.45) | 0.16 (–0.12, 0.43) | –0.20 (–0.38,–0.01) | 0.07 (0.00, 0.15) | 0.00 (–0.02, 0.03) | 0.00 (–0.01, 0.01) | 0.06 (0.03, 0.09) | |
| Model 2 | –0.27 (–1.37, 0.83) | 0.05 (–0.29, 0.39) | –0.27 (–0.47,–0.06) | 0.00 (–0.08, 0.08) | –0.08 (–0.12,–0.04) | –0.07 (–0.09,–0.05) | –0.02 (–0.06, 0.03) | |
| Model 3 | –0.16 (–1.25, 0.93) | 0.08 (–0.26, 0.42) | –0.22 (–0.43,–0.02) | 0.02 (–0.05, 0.10) | –0.05 (–0.09,–0.01) | –0.05 (–0.07,–0.03) | –0.02 (–0.07, 0.03) | |
| Model 4 | –0.15 (–1.24, 0.94) | 0.08 (–0.26, 0.42) | –0.22 (–0.43,–0.02) | 0.03 (–0.05, 0.10) | –0.05 (–0.09,–0.01) | –0.05 (–0.07,–0.03) | –0.02 (–0.07, 0.03) | |
| Model 5 | –0.21 (–0.88, 0.46) | 0.13 (–0.15, 0.40) | –0.23 (–0.42,–0.04) | 0.03 (–0.04, 0.11) | –0.05 (–0.09,–0.02) | –0.05 (–0.07,–0.02) | –0.02 (–0.07, 0.03) | |
|  |  |  |  |  |  |  |  | |
| **18 years** |  |  |  |  |  |  |  | |
| Model 1 | 0.08 (–0.86, 1.01) | 0.02 (–0.37, 0.41) | –0.11 (–0.36, 0.14) | 0.09 (–0.01, 0.19) | 0.00 (–0.03, 0.04) | –0.03 (–0.04,–0.01) | –0.03 (–0.07, 0.01) | |
| Model 2 | 0.02 (–1.26, 1.31) | –0.08 (–0.49, 0.34) | –0.20 (–0.44, 0.04) | 0.01 (–0.09, 0.10) | –0.08 (–0.12,–0.03) | –0.10 (–0.12,–0.07) | –0.10 (–0.16,–0.04) | |
| Model 3 | 0.07 (–1.20, 1.34) | –0.06 (–0.47, 0.35) | –0.17 (–0.41, 0.07) | 0.03 (–0.06, 0.13) | –0.06 (–0.10,–0.01) | –0.08 (–0.10,–0.06) | –0.11 (–0.17,–0.05) | |
| Model 4 | 0.07 (–1.20, 1.34) | –0.05 (–0.46, 0.36) | –0.17 (–0.41, 0.07) | 0.04 (–0.06, 0.13) | –0.05 (–0.10,–0.01) | –0.08 (–0.10,–0.06) | –0.11 (–0.17,–0.05) | |
| Model 5 | 0.06 (–0.77, 0.89) | –0.05 (–0.40, 0.30) | –0.16 (–0.39, 0.06) | 0.04 (–0.06, 0.13) | –0.05 (–0.10,–0.01) | –0.08 (–0.10,–0.05) | –0.10 (–0.16,–0.04) | |
|  |  |  |  |  |  |  |  | |

***Model 1****: Unadjusted;* ***Model 2****: Adjusted for maternal age, education, household income, and IPW and clustering effect;* ***Model 3****: Model 2 plus smoking during pregnancy, gestational diabetes, and gestational hypertension (included in the manuscript);* ***Model 4****: Model 3 without IPW;* ***Model 5****: Model 3 without clustering-effect*

| **Supplementary Table S4**. Predicted mean BMI z–score by category of gestational age in girls (95% CI) (*n = 30 842*) | | | | | | | |  |
| --- | --- | --- | --- | --- | --- | --- | --- | --- |
|  | **Extremely Preterm**  **23–27 weeks** | **Very Preterm**  **28–31 weeks** | **Moderately Preterm**  **32–33 weeks** | **Late Preterm**  **34–36 weeks** | **Early Term**  **37–38 weeks** | **Term**  **39–41 weeks** | **Post Term**  **42–43 weeks** | |
| **Birth** | | | | | | | | |
| Model 1 | –4.35 (–4.77,–3.93) | –3.28 (–3.49,–3.07) | –2.36 (–2.53,–3.07) | –1.13 (–1.19,–1.07) | –0.31 (–0.34,–0.28) | 0.10 (0.09, 0.12) | 0.27 (0.23, 0.31) | |
| Model 2 | –5.08 (–5.71,–4.45) | –4.05 (–4.29,–3.80) | –3.06 (–3.23,–2.849 | –1.66 (–1.72,–1.60) | –0.60 (–0.63,–0.57) | 0.07 (0.05, 0.09) | 0.44 (0.40, 0.48) | |
| Model 3 | –5.04 (–5.67,–4.41) | –4.00 (–4.24,–3.76) | –3.01 (–3.18,–2.84) | –1.62 (–1.68,–1.56) | –0.56 (–0.59,–0.53) | 0.10 (0.08, 0.12) | 0.43 (0.40, 0.47) | |
| Model 4 | –5.02 (–5.65,–4.41) | –4.00 (–4.25,–3.76) | –3.00 (–3.18,–2.83) | –1.63 (–1.68,–1.56) | –0.56 (–0.59,–0.53) | 0.10 (0.08, 0.12) | 0.44 (0.40, 0.47) | |
| Model 5 | –5.01 (–5.42,–4.60) | –4.02 (–4.22,–3.81) | –3.01 (–3.17,–2.85) | –1.63 (–1.69,–1.57) | –0.57 (–0.59,–0.54) | 0.10 (0.07, 0.11) | 0.43 (0.39, 0.46) | |
|  |  |  |  |  |  |  |  | |
| **5 months** | | | | | | | | |
| Model 1 | –1.30 (–1.81,–0.79) | –0.61 (–0.87,–0.36) | –0.24 (–0.43,–0.04) | –0.06 (–0.13, 0.01) | –0.02 (–0.06, 0.01) | 0.00 (–0.01, 0.02) | 0.05 (0.01, 0.09) | |
| Model 2 | –1.15 (–1.94,–0.36) | –0.68 (–0.97,–0.39) | –0.32 (–0.52,–0.12) | –0.14 (–0.21,–0.07) | –0.11 (–0.14,–0.07) | –0.08 (–0.10,–0.06) | –0.04 (–0.08,–0.00) | |
| Model 3 | –1.09 (–1.88,–0.30) | –0.64 (–0.94,–0.35) | –0.27 (–0.47,–0.07) | –0.10 (–0.16,–0.03) | –0.07 (–0.10,–0.04) | –0.06 (–0.07,–0.03) | –0.04 (–0.08,–0.00) | |
| Model 4 | –1.06 (–1.85,–0.27) | –0.65 (–0.94,–0.35) | –0.27 (–0.46,–0.07) | –0.09 (–0.16,–0.03) | –0.07 (–0.10,–0.04) | –0.05 (–0.07,–0.03) | –0.04 (–0.08,–0.00) | |
| Model 5 | –1.33 (–1.83,–0.84) | –0.63 (–0.94,–0.38) | –0.26 (–0.45,–0.08) | –0.10 (–0.16,–0.03) | –0.07 (–0.10,–0.03) | –0.05 (–0.07,–0.03) | –0.04 (–0.08,–0.00) | |
|  |  |  |  |  |  |  |  | |
| **1 year** | | | | | | | | |
| Model 1 | –0.92 (–1.39,–0.44) | –0.64 (–0.88,–0.39) | –0.18 (–0.37, 0.00) | –0.01 (–0.07, 0.06) | –0.01 (–0.05, 0.02) | –0.00 (–0.02, 0.01) | 0.04 (0.00, 0.08) | |
| Model 2 | –0.87 (–1.61,–0.12) | –0.72 (–1.01,–0.44) | –0.29 (–0.49,–0.08) | –0.10 (–0.16,–0.03) | –0.10 (–0.13,–0.06) | –0.08 (–0.10,–0.01) | –0.04 (–0.09, 0.01) | |
| Model 3 | –0.81 (–1.55,–0.07) | –0.70 (–0.98,–0.42) | –0.24 (–0.44,–0.04) | –0.06 (–0.12,–0.01) | –0.06 (–0.09,–0.03) | –0.06 (–0.08,–0.04) | –0.05 (–0.09, 0.01) | |
| Model 4 | –0.80 (–1.54,–0.06) | –0.71 (–0.99,–0.42) | –0.23 (–0.44,–0.04) | –0.05 (–0.12,–0.01) | –0.06 (–0.09,–0.03) | –0.06 (–0.07,–0.04) | –0.04 (–0.09, 0.00) | |
| Model 5 | –0.98 (–1.44,–0.52) | –0.69 (–0.93,–0.45) | –0.23 (–0.41,–0.04) | –0.05 (–0.12,–0.01) | –0.06 (–0.09,–0.03) | –0.06 (–0.08,–0.04) | –0.05 (–0.09, 0.01) | |
|  |  |  |  |  |  |  |  | |
| **7 years** | | | | | | | | |
| Model 1 | –0.64 (–1.22,–0.06) | –0.29 (–0.54,–0.04) | –0.10 (–0.31,–0.10) | –0.09 (–0.16,–0.02) | –0.01 (–0.03, 0.02) | –0.00 (–0.01, 0.01) | 0.05 (0.01, 0.09) | |
| Model 2 | –0.72 (–1.63, 1.19) | –0.37 (–0.67,–0.07) | –0.20 (–0.41,–0.02) | –0.17 (–0.24,–0.09) | –0.09 (–0.13,–0.06) | –0.08 (–0.10,–0.06) | –0.03 (–0.08, 0.01) | |
| Model 3 | –0.73 (–1.63, 1.17) | –0.32 (–0.62,–0.03) | –0.14 (–0.36,–0.07) | –0.13 (–0.21,–0.05) | –0.05 (–0.09,–0.02) | –0.05 (–0.07,–0.04) | –0.03 (–0.08, 0.01) | |
| Model 4 | –0.70 (–1.60, 1.20) | –0.33 (–0.62,–0.03) | –0.14 (–0.35,–0.07) | –0.12 (–0.20,–0.05) | –0.05 (–0.09,–0.01) | –0.05 (–0.07,–0.03) | –0.03 (–0.07, 0.01) | |
| Model 5 | –0.71 (–1.27,–0.14) | –0.29 (–0.54,–0.04) | –0.13 (–0.33,–0.06) | –0.13 (–0.20,–0.05) | –0.05 (–0.08,–0.01) | –0.05 (–0.07,–0.03) | –0.03 (–0.07, 0.01) | |
|  |  |  |  |  |  |  |  | |
| **11 years** | | | | | | | | |
| Model 1 | –0.44 (–1.08, 0.21) | –0.07 (–0.37, 0.23) | –0.08 (–0.29, 0.13) | –0.01 (–0.08, 0.07) | 0.01 (–0.00, 0.02) | 0.01 (–0.00, 0.02) | 0.05 (0.01, 0.08) | |
| Model 2 | –0.52 (–1.54, 0.50) | –0.16 (–0.52, 0.20) | –0.17 (–0.40, 0.06) | –0.08 (–0.16, 0.00) | –0.06 (–0.10, 0.02) | –0.08 (–0.10,–0.07) | –0.03 (–0.08, 0.01) | |
| Model 3 | –0.47 (–1.48, 0.54) | –0.11 (–0.47, 0.24) | –0.12 (–0.35, 0.16) | –0.04 (–0.12, 0.04) | –0.02 (–0.05, 0.02) | –0.05 (–0.07,–0.03) | –0.03 (–0.08, 0.01) | |
| Model 4 | –0.44 (–1.45, 0.58) | –0.11 (–0.47, 0.24) | –0.12 (–0.35, 0.10) | –0.04 (–0.12, 0.04) | –0.02 (–0.05, 0.02) | –0.05 (–0.07,–0.03) | –0.03 (–0.08, 0.01) | |
| Model 5 | –0.43 (–1.48, 0.21) | –0.08 (–0.38, 0.22) | –0.11 (–0.32, 0.10) | –0.04 (–0.11, 0.04) | –0.01 (–0.05, 0.03) | –0.04 (–0.06,–0.02) | –0.03 (–0.08, 0.02) | |
|  |  |  |  |  |  |  |  | |
| **18 years** | | | | | | | | |
| Model 1 | –0.38 (–1.01, 0.25) | –0.13 (–0.46, 0.20) | 0.05 (–0.21, 0.31) | 0.04 (–0.05, 0.13) | –0.01 (–0.04, 0.01) | –0.02 (–0.03,–0.01) | 0.04 (0.01, 0.08) | |
| Model 2 | –0.43 (–1.24, 0.39) | –0.20 (–0.55, 0.14) | 0.05 (–0.30, 0.20) | –0.05 (–0.13, 0.04) | –0.09 (–0.13,–0.05) | –0.10 (–0.12,–0.08) | –0.04 (–0.09, 0.01) | |
| Model 3 | –0.36 (–1.15, 0.44) | –0.16 (–0.49, 0.18) | 0.02 (–0.23, 0.26) | –0.01 (–0.09, 0.07) | –0.05 (–0.09,–0.01) | –0.08 (–0.10,–0.05) | –0.04 (–0.09, 0.01) | |
| Model 4 | –0.34 (–1.14, 0.46) | –0.16 (–0.50, 0.18) | 0.02 (–0.22, 0.26) | –0.01 (–0.09, 0.07) | –0.05 (–0.09,–0.01) | –0.08 (–0.10,–0.05) | –0.04 (–0.09, 0.01) | |
| Model 5 | –0.42 (–0.99, 0.14) | –0.14 (–0.43, 0.15) | 0.02 (–0.22, 0.25) | –0.01 (–0.09, 0.08) | –0.05 (–0.09,–0.01) | –0.07 (–0.09,–0.05) | –0.04 (–0.09, 0.02) | |
|  |  |  |  |  |  |  |  | |

***Model 1****: Unadjusted;* ***Model 2****: Adjusted for maternal age, education, household income, and IPW and clustering effect;* ***Model 3****: Model 2 plus smoking during pregnancy, gestational diabetes, and gestational hypertension (included in the manuscript);* ***Model 4****: Model 3 without IPW;* ***Model 5****: Model 3 without clustering-effect*

| **Supplementary Table S5.** Predicted mean birth length and mean height z–score in boys, by category of gestational age (95% CI) (*n = 31 407*) | | | | | | | |  |
| --- | --- | --- | --- | --- | --- | --- | --- | --- |
|  | **Extremely Preterm**  **23–27 weeks** | **Very Preterm**  **28–31 weeks** | **Moderately Preterm**  **32–33 weeks** | **Late Preterm**  **34–36 weeks** | **Early Term**  **37–38 weeks** | **Term**  **39–41 weeks** | **Post Term**  **42–43 weeks** | |
| **Birth** | | | | | | | | |
| Model 1 | –6.56 (–7.03,–6.08) | –4.39 (–4.59,–4.19) | –2.92 (–3.06,–2.78) | –1.46 (–1.52,–1.41) | –0.49 (–0.52,–0.47) | 0.15 (0.14, 0.16) | 0.55 (0.52, 0.59) | |
| Model 2 | –6.45 (–7.15,–5.75) | –4.36 (–4.59,–4.13) | –2.85 (–3.00,–2.70) | –1.42 (–1.47,–1.36) | –0.45 (–0.48,–0.42) | 0.19 (0.16, 0.21) | 0.59 (0.55, 0.63) | |
| Model 3 | –6.40 (–7.10,–5.72) | –4.31 (–4.53,–4.09) | –2.83 (–2.97,–2.68) | –1.39 (–1.44,–1.33) | –0.44 (–0.47,–0.41) | 0.17 (0.15, 0.19) | 0.55 (0.52, 0.59) | |
| Model 4 | –6.40 (–7.10,–5.72) | –4.31 (–4.53,–4.09) | –2.82 (–2.97,–2.68) | –1.39 (–1.44,–1.33) | –0.44 (–0.47,–0.41) | 0.17 (0.15, 0.19) | 0.55 (0.52, 0.59) | |
| Model 5 | –6.43 (–7.10,–5.72) | –4.30 (–4.53,–4.09) | –2.85 (–2.97,–2.68) | –1.39 (–1.44,–1.33) | –0.44 (–0.47,–0.41) | 0.17 (0.15, 0.19) | 0.55 (0.52, 0.59) | |
|  |  |  |  |  |  |  |  | |
| **5 months** | | | | | | | | |
| Model 1 | –2.77 (–3.50,–2.05) | –2.50 (–2.72,–2.28) | –1.67 (–1.83,–1.52) | –0.74 (–0.80,–0.68) | –0.27 (–0.30,–0.24) | 0.08 (0.07, 0.09) | 0.30 (0.27, 0.34) | |
| Model 2 | –2.78 (–3.95,–1.61) | –2.43 (–2.70,–2.17) | –1.61 (–1.78,–1.44) | –0.70 (–0.76,–0.64) | –0.23 (–0.26,–0.20) | 0.11 (0.09, 0.13) | 0.34 (0.30, 0.38) | |
| Model 3 | –2.73 (–3.89,–1.57) | –2.37 (–2.63,–2.11) | –1.58 (–1.74,–1.42) | –0.67 (–0.73,–0.61) | –0.22 (–0.25,–0.19) | 0.10 (0.08, 0.12) | 0.30 (0.26, 0.34) | |
| Model 4 | –2.73 (–3.89,–1.57) | –2.37 (–2.63,–2.11) | –1.58 (–1.74,–1.42) | –0.67 (–0.74,–0.61) | –0.22 (–0.25,–0.19) | 0.10 (0.08, 0.12) | 0.30 (0.26, 0.34) | |
| Model 5 | –2.66 (–3.89,–1.57) | –2.40 (–2.63,–2.11) | –1.60 (–1.74,–1.42) | –0.67 (–0.73,–0.61) | –0.22 (–0.25,–0.19) | 0.10 (0.08, 0.12) | 0.30 (0.26, 0.34) | |
|  |  |  |  |  |  |  |  | |
| **1 year** | | | | | | | | |
| Model 1 | –1.27 (–1.80,–0.73) | –0.91 (–1.13,–0.69) | –0.64 (–0.80,–0.49) | –0.22 (–0.28,–0.16) | –0.10 (–0.13,–0.08) | 0.02 (0.01, 0.04) | 0.17 (0.14, 0.21) | |
| Model 2 | –1.19 (–1.99,–0.39) | –0.87 (–1.13,–0.62) | –0.59 (–0.76,–0.42) | –0.19 (–0.24,–0.12) | –0.07 (–0.10,–0.03) | 0.06 (0.04, 0.08) | 0.20 (0.16, 0.24) | |
| Model 3 | –1.18 (–1.97,–0.39) | –0.81 (–1.07,–0.56) | –0.56 (–0.73,–0.40) | –0.15 (–0.21,–0.09) | –0.06 (–0.09,–0.03) | 0.04 (0.02, 0.06) | 0.16 (0.13, 0.20) | |
| Model 4 | –1.18 (–1.96,–0.39) | –0.81 (–1.07,–0.56) | –0.56 (–0.72,–0.40) | –0.15 (–0.21,–0.09) | –0.08 (–0.09,–0.03) | 0.04 (0.02, 0.06) | 0.17 (0.13, 0.21) | |
| Model 5 | –1.16 (–1.97,–0.39) | –0.81 (–1.07,–0.56) | –0.57 (–0.73,–0.40) | –0.14 (–0.21,–0.09) | –0.06 (–0.09,–0.03) | 0.04 (0.02, 0.06) | 0.17 (0.13, 0.20) | |
|  |  |  |  |  |  |  |  | |
| **7 years** | | | | | | | | |
| Model 1 | –0.97 (–1.50,–0.45) | –0.25 (–0.48,–0.01) | –0.34 (–0.50,–0.17) | –0.11 (–0.18,–0.05) | –0.04 (–0.07,–0.02) | 0.00 (–0.00, 0.01) | 0.08 (0.05, 0.11) | |
| Model 2 | –0.87 (–1.66,–0.07) | –0.19 (–0.46, 0.07) | –0.31 (–0.49,–0.13) | –0.08 (–0.14,–0.01) | –0.01 (–0.04, 0.03) | 0.04 (0.02, 0.06) | 0.11 (0.07, 0.15) | |
| Model 3 | –0.80 (–1.58,–0.02) | –0.14 (–0.40, 0.11) | –0.28 (–0.45,–0.11) | –0.05 (–0.11,–0.02) | 0.00 (–0.03, 0.04) | 0.02 (0.00, 0.04) | 0.07 (0.03, 0.11) | |
| Model 4 | –0.80 (–1.58,–0.02) | –0.14 (–0.40, 0.12) | –0.28 (–0.45,–0.11) | –0.05 (–0.11,–0.02) | 0.00 (–0.03, 0.03) | 0.02 (0.00, 0.04) | 0.07 (0.03, 0.11) | |
| Model 5 | –0.84 (–1.33,–0.34) | –0.15 (–0.37, 0.07) | –0.26 (–0.42,–0.10) | –0.05 (–0.11, 0.02) | 0.00 (–0.03, 0.04) | 0.02 (0.00, 0.04) | 0.08 (0.04, 0.12) | |
|  |  |  |  |  |  |  |  | |
| **11 years** | | | | | | | | |
| Model 1 | –0.79 (–1.44,–0.14) | –0.19 (–0.45, 0.07) | –0.33 (–0.51,–0.15) | –0.07 (–0.14,–0.01) | –0.01 (–0.04, 0.01) | 0.00 (–0.01, 0.01) | 0.03 (0.00, 0.07) | |
| Model 2 | –0.70 (–1.71, 0.31) | –0.15 (–0.47, 0.17) | –0.28 (–0.48,–0.09) | –0.03 (–0.11, 0.04) | 0.03 (–0.01, 0.06) | 0.03 (–0.01, 0.06) | 0.07 (–0.02, 0.11) | |
| Model 3 | –0.65 (–1.64, 0.33) | –0.11 (–0.41, 0.19) | –0.26 (–0.44,–0.07) | –0.01 (–0.08, 0.06) | 0.03 (–0.00, 0.07) | 0.02 (–0.00, 0.03) | 0.03 (–0.02, 0.07) | |
| Model 4 | –0.65 (–1.63, 0.33) | –0.10 (–0.41, 0.20) | –0.25 (–0.44,–0.06) | –0.00 (–0.08, 0.07) | 0.03 (–0.00, 0.07) | 0.02 (–0.00, 0.43) | 0.03 (–0.02, 0.07) | |
| Model 5 | –0.66 (–1.29,–0.03) | –0.10 (–0.36, 0.15) | –0.26 (–0.43,–0.08) | –0.01 (–0.08, 0.07) | 0.03 (–0.00, 0.07) | 0.02 (–0.00, 0.04) | 0.03 (–0.02, 0.07) | |
|  |  |  |  |  |  |  |  | |
| **18 years** | | | | | | | | |
| Model 1 | –0.53 (–1.42, 0.36) | –0.52 (–0.88,–0.16) | –0.15 (–0.39, 0.09) | –0.14 (–0.24,–0.05) | –0.01 (–0.04, 0.02) | 0.01 (0.00, 0.03) | 0.05 (0.01, 0.09) | |
| Model 2 | –0.59 (–1.77, 0.60) | –0.41 (–0.79,–0.04) | –0.14 (–0.37, 0.09) | –0.09 (–0.19,–0.00) | 0.03 (–0.01, 0.07) | 0.05 (0.03, 0.08) | 0.09 (0.03, 0.14) | |
| Model 3 | –0.53 (–1.67, 0.62) | –0.35 (–0.71, 0.01) | –0.13 (–0.35, 0.09) | –0.07 (–0.16, 0.02) | 0.04 (-0.00, 0.08) | 0.03 (0.01, 0.06) | 0.05 (–0.01, 0.10) | |
| Model 4 | –0.53 (–1.67, 0.62) | –0.35 (–0.71, 0.02) | –0.12 (–0.35, 0.10) | –0.07 (–0.15, 0.02) | 0.04 (-0.00, 0.08) | 0.04 (0.01, 0.06) | 0.05 (–0.01, 0.10) | |
| Model 5 | –0.44 (–1.67, 0.62) | –0.38 (–0.70,–0.07) | –0.12 (–0.33, 0.09) | –0.07 (–0.16, 0.02) | 0.04 (-0.00, 0.08) | 0.03 (0.01, 0.06) | 0.05 (–0.01, 0.10) | |
|  |  |  |  |  |  |  |  | |

***Model 1****: Unadjusted;* ***Model 2****: Adjusted for maternal age, education, household income, and IPW and clustering effect;* ***Model 3****: Model 2 plus smoking during pregnancy, gestational diabetes, and gestational hypertension (included in the manuscript);* ***Model 4****: Model 3 without IPW;* ***Model 5****: Model 3 without clustering-effect*

| **Supplementary Table S6.** Predicted mean birth length and mean height z–score in girls, by category of gestational age (95% CI) (*n = 31 218*) | | | | | | | |  |
| --- | --- | --- | --- | --- | --- | --- | --- | --- |
|  | **Extremely Preterm**  **23–27 weeks** | **Very Preterm**  **28–31 weeks** | **Moderately Preterm**  **32–33 weeks** | **Late Preterm**  **34–36 weeks** | **Early Term**  **37–38 weeks** | **Term**  **39–41 weeks** | **Post Term**  **42–43 weeks** | |
| **Birth** | | | | | | | | |
| Model 1 | –6.72 (–7.11,–6.32) | –4.80 (–5.00,–4.60) | –3.03 (–3.20,–2.88) | –1.52 (–1.58,–1.46) | –0.50 (–0.53,–0.47) | 0.14 (0.13, 0.15) | 0.53 (0.50, 0.56) | |
| Model 2 | –6.71 (–7.28,–6.15) | –4.71 (–4.94,–4.49) | –3.00 (–3.17,–2.84) | –1.47 (–1.53,–1.41) | –0.45 (–0.48,–0.43) | 0.18 (0.17, 0.20) | 0.57 (0.53, 0.61) | |
| Model 3 | –6.69 (–7.25,–6.13) | –4.64 (–4.87,–4.42) | –2.91 (–3.07,–2.75) | –1.42 (–1.48,–1.36) | –0.42 (–0.45,–0.39) | 0.18 (0.16, 0.20) | 0.54 (0.50, 0.57) | |
| Model 4 | –6.70 (–7.26,–6.14) | –4.66 (–4.88,–4.44) | –2.91 (–3.07,–2.75) | –1.42 (–1.48,–1.36) | –0.42 (–0.45,–0.39) | 0.18 (0.16, 0.20) | 0.54 (0.50, 0.58) | |
| Model 5 | –6.66 (–7.05,–6.28) | –4.66 (–4.85,–4.46) | –2.90 (–3.05,–2.74) | –1.42 (–1.48,–1.37) | –0.43 (–0.46,–0.40) | 0.18 (0.16, 0.20) | 0.54 (0.50, 0.57) | |
|  |  |  |  |  |  |  |  | |
| **5 months** | | | | | | | | |
| Model 1 | –3.01 (–3.50,–2.53) | –2.27 (–2.51,–2.04) | –1.60 (–1.78,–1.42) | –0.76 (–0.83,–0.70) | –0.27 (–0.30,–0.24) | 0.07 (0.05, 0.08) | 0.28 (0.24,0.32) | |
| Model 2 | –2.67 (–3.41,–1.93) | –2.22 (–2.50,–1.95) | –1.56 (–1.75,–1.37) | –0.72 (–0.78,–0.65) | –0.22 (–0.26,–0.19) | 0.11 (0.09, 0.13) | 0.32 (0.27, 0.36) | |
| Model 3 | –2.63 (–3.36,–1.90) | –2.16 (–2.43,–1.90) | –1.46 (–1.65,–1.28) | –0.67 (–0.73,–0.60) | –0.19 (–0.22,–0.16) | 0.10 (0.08, 0.12) | 0.29 (0.25, 0.33) | |
| Model 4 | –2.63 (–3.36,–1.90) | –2.18 (–2.45,–1.90) | –1.46 (–1.65,–1.28) | –0.67 (–0.73,–0.60) | –0.19 (–0.22,–0.16) | 0.10 (0.08, 0.12) | 0.29 (0.25, 0.33) | |
| Model 5 | –2.93 (–3.41,–2.47) | –2.14 (–2.37,–1.90) | –1.46 (–1.63,–1.28) | –0.67 (–0.73,–0.60) | –0.19 (–0.22,–0.16) | 0.10 (0.08, 0.12) | 0.29 (0.25, 0.33) | |
|  |  |  |  |  |  |  |  | |
| **1 year** | | | | | | | | |
| Model 1 | –1.54 (–1.99,–1.09) | –1.17 (–1.39,–0.94) | –0.85 (–1.03,–0.67) | –0.34 (–0.41,–0.28) | –0.14 (–0.16,–0.11) | 0.03 (0.02, 0.04) | 0.17 (0.14,0.21) | |
| Model 2 | –1.53 (–2.21,–0.85) | –1.13 (–1.40,–0.87) | –0.80 (–0.98,–0.60) | –0.30 (–0.36,–0.23) | –0.10 (–0.13,–0.06) | 0.07 (0.05, 0.09) | 0.21 (0.17, 0.25) | |
| Model 3 | –1.50 (–2.17,–0.82) | –1.07 (–1.33,–0.81) | –0.70 (–0.88,–0.51) | –0.24 (–0.31,–0.18) | –0.06 (–0.10,–0.03) | 0.06 (0.04, 0.08) | 0.18 (0.14, 0.22) | |
| Model 4 | –1.50 (–2.17,–0.82) | –1.09 (–1.35,–0.83) | –0.70 (–0.88,–0.51) | –0.24 (–0.31,–0.18) | –0.06 (–0.10,–0.03) | 0.06 (0.04, 0.08) | 0.18 (0.14, 0.22) | |
| Model 5 | –1.47 (–1.91,–1.03) | –1.03 (–1.25,–0.80) | –0.71 (–0.88,–0.54) | –0.24 (–0.31,–0.18) | –0.06 (–0.09,–0.03) | 0.06 (0.05, 0.09) | 0.18 (0.14, 0.22) | |
|  |  |  |  |  |  |  |  | |
| **7 years** | | | | | | | | |
| Model 1 | –0.77 (–1.32,–0.22) | –0.42 (–0.66,–0.18) | –0.43 (–0.62,–0.24) | –0.15 (–0.22,–0.08) | –0.05 (–0.08,–0.02) | 0.01 (–0.00, 0.02) | 0.07 (0.03,0.10) | |
| Model 2 | –0.82 (–1.66, 0.01) | –0.35 (–0.62,–0.07) | –0.38 (–0.59,–0.18) | –0.10 (–0.18, 0.03) | –0.01 (–0.04, 0.03) | 0.04 (0.02, 0.07) | 0.10 (0.05, 0.15) | |
| Model 3 | –0.86 (–1.68,–0.04) | –0.29 (–0.55,–0.02) | –0.29 (–0.49,–0.10) | –0.06 (–0.13, 0.01) | 0.03 (–0.01, 0.06) | 0.04 (0.02, 0.06) | 0.07 (0.03, 0.11) | |
| Model 4 | –0.86 (–1.68,–0.03) | –0.31 (–0.58,–0.04) | –0.29 (–0.49,–0.09) | –0.06 (–0.13, 0.02) | 0.03 (–0.01, 0.06) | 0.04 (0.02, 0.06) | 0.07 (0.03, 0.12) | |
| Model 5 | –0.75 (–1.29,–0.22) | –0.27 (–0.49,–0.04) | –0.29 (–0.48,–0.10) | –0.05 (–0.12, 0.02) | 0.03 (–0.01, 0.06) | 0.04 (0.02, 0.06) | 0.07 (0.03, 0.12) | |
|  |  |  |  |  |  |  |  | |
| **11 years** | | | | | | | | |
| Model 1 | –0.44 (–1.03, 0.16) | –0.24 (–0.52, 0.04) | –0.32 (–0.52,–0.11) | –0.16 (–0.23,–0.09) | –0.05 (–0.07,–0.02) | 0.00 (–0.01, 0.01) | 0.05 (0.02,0.09) | |
| Model 2 | –0.51 (–1.42, 0.39) | –0.13 (–0.47, 0.20) | –0.28 (–0.50, 0.05) | –0.12 (–0.20,–0.05) | 0.01 (–0.04, 0.03) | 0.04 (0.02, 0.06) | 0.09 (0.04, 0.13) | |
| Model 3 | –0.52 (–1.41, 0.36) | –0.07 (–0.39, 0.25) | –0.20 (–0.40, 0.02) | –0.08 (–0.15,–0.01) | 0.02 (–0.02, 0.06) | 0.04 (0.02, 0.06) | 0.06 (0.02, 0.09) | |
| Model 4 | –0.51 (–1.40, 0.38) | –0.10 (–0.43, 0.22) | –0.19 (–0.40, 0.02) | –0.08 (–0.15,–0.00) | 0.02 (–0.01, 0.06) | 0.04 (0.02, 0.06) | 0.06 (0.02, 0.10) | |
| Model 5 | –0.43 (–1.01, 0.15) | –0.08 (–0.36, 0.20) | –0.18 (–0.39, 0.01) | –0.07 (–0.15,–0.00) | 0.03 (–0.01, 0.06) | 0.04 (0.02, 0.06) | 0.06 (0.01, 0.10) | |
|  |  |  |  |  |  |  |  | |
| **18 years** | | | | | | | | |
| Model 1 | –0.93 (–1.38,–0.18) | –0.44 (–0.76,–0.13) | –0.43 (–0.67,–0.19) | –0.13 (–0.22,–0.05) | –0.03 (–0.06,–0.01) | 0.03 (0.02, 0.04) | 0.00 (–0.03,0.04) | |
| Model 2 | –0.93 (–1.67,–0.18) | –0.31 (–0.63, 0.02) | –0.38 (–0.61,–0.14) | –0.09 (–0.17, 0.00) | 0.01 (–0.03, 0.05) | 0.07 (0.05, 0.09) | 0.05 (–0.00, 0.10) | |
| Model 3 | –0.93 (–1.65,–0.22) | –0.22 (–0.53, 0.09) | –0.29 (–0.51,–0.06) | –0.04 (–0.12, 0.04) | 0.04 (0.00, 0.08) | 0.06 (0.04, 0.08) | 0.02 (–0.03, 0.07) | |
| Model 4 | –0.92 (–1.64,–0.20) | –0.26 (–0.57, 0.05) | –0.29 (–0.51,–0.06) | –0.04 (–0.12, 0.04) | 0.04 (0.00, 0.08) | 0.06 (0.04, 0.09) | 0.02 (–0.03, 0.07) | |
| Model 5 | –0.77 (–1.29,–0.25) | –0.24 (–0.51, 0.03) | –0.29 (–0.50,–0.07) | –0.04 (–0.12, 0.03) | 0.04 (0.00, 0.08) | 0.06 (0.04, 0.08) | 0.02 (–0.03, 0.06) | |
|  |  |  |  |  |  |  |  | |

***Model 1****: Unadjusted;* ***Model 2****: Adjusted for maternal age, education, household income, and IPW and clustering effect;* ***Model 3****: Model 2 plus smoking during pregnancy, gestational diabetes, and gestational hypertension (included in the manuscript);* ***Model 4****: Model 3 without IPW;* ***Model 5****: Model 3 without clustering-effect*
